# Identification of key factors for malnutrition diagnosis in chronic gastrointestinal diseases using machine learning underscores the importance of GLIM criteria as well as additional parameters

**DOI:** 10.3389/fnut.2024.1479501

**Published:** 2024-12-12

**Authors:** Karen Rischmüller, Vanessa Caton, Markus Wolfien, Luise Ehlers, Matti van Welzen, David Brauer, Lea F. Sautter, Fatuma Meyer, Luzia Valentini, Mats L. Wiese, Ali A. Aghdassi, Robert Jaster, Olaf Wolkenhauer, Georg Lamprecht, Saptarshi Bej

**Affiliations:** ^1^Division of Gastroenterology and Endocrinology, Department of Internal Medicine II, Rostock University Medical Center, Rostock, Germany; ^2^Department of Systems Biology and Bioinformatics, Institute of Computer Science, University of Rostock, Rostock, Germany; ^3^Faculty of Medicine Carl Gustav Carus, Institute for Medical Informatics and Biometry, TUD Dresden University of Technology, Dresden, Germany; ^4^Center for Scalable Data Analytics and Artificial Intelligence (ScaDS.AI) Dresden/Leipzig, Dresden, Germany; ^5^Department of Agriculture and Food Sciences, Neubrandenburg Institute of Evidence-Based Nutrition (NIED), University of Applied Sciences Neubrandenburg, Neubrandenburg, Germany; ^6^Department of Medicine A, University Medicine Greifswald, Greifswald, Germany; ^7^Leibniz-Institute for Food Systems Biology, Technical University of Munich, Freising, Germany; ^8^Indian Institute of Science Education and Research, Thiruvananthapuram, India

**Keywords:** malnutrition, GLIM criteria, machine learning, supervised and unsupervised learning, decision trees, gastrointestinal diseases, liver cirrhosis

## Abstract

**Introduction:**

Disease-related malnutrition is common but often underdiagnosed in patients with chronic gastrointestinal diseases, such as liver cirrhosis, short bowel and intestinal insufficiency, and chronic pancreatitis. To improve malnutrition diagnosis in these patients, an evaluation of the current Global Leadership Initiative on Malnutrition (GLIM) diagnostic criteria, and possibly the implementation of additional criteria, is needed.

**Aim:**

This study aimed to identify previously unknown and potentially specific features of malnutrition in patients with different chronic gastrointestinal diseases and to validate the relevance of the GLIM criteria for clinical practice using machine learning (ML).

**Methods:**

Between 10/2018 and 09/2021, *n* = 314 patients and controls were prospectively enrolled in a cross-sectional study. A total of *n* = 230 features (anthropometric data, body composition, handgrip strength, gait speed, laboratory values, dietary habits, physical activity, mental health) were recorded. After data preprocessing (cleaning, feature exploration, imputation of missing data), *n* = 135 features were included in the ML analyses. Supervised ML models were used to classify malnutrition, and key features were identified using SHapley Additive exPlanations (SHAP).

**Results:**

Supervised ML effectively classified malnourished versus non-malnourished patients and controls. Excluding the existing GLIM criteria and malnutrition risk reduced model performance (sensitivity -19%, specificity -8%, F1-score -10%), highlighting their significance. Besides some GLIM criteria (weight loss, reduced food intake, disease/inflammation), additional anthropometric (hip and upper arm circumference), body composition (phase angle, SMMI), and laboratory markers (albumin, pseudocholinesterase, prealbumin) were key features for malnutrition classification.

**Conclusion:**

ML analysis confirmed the clinical applicability of the current GLIM criteria and identified additional features that may improve malnutrition diagnosis and understanding of the pathophysiology of malnutrition in chronic gastrointestinal diseases.

## Introduction

Disease-related malnutrition is a common but often underestimated complication in hospitalized patients with a particularly high prevalence of more than 30% in gastrointestinal diseases (1). Major factors leading to disease-related malnutrition include decreased food intake despite increased energy and protein requirements, and stress-induced catabolism due to inflammation (2). Disease-related malnutrition increases the risk of infections, organ dysfunction, and impaired healing resulting in prolonged hospitalization, decreased functional status, impaired quality of life, and ultimately increased morbidity and mortality (2, 3). Therefore, the early detection and treatment of malnutrition is of great importance but requires effective diagnostic markers and a better understanding of its underlying mechanisms.

The Global Leadership Initiative on Malnutrition (GLIM) has defined an algorithm for the operational diagnosis of malnutrition. First, the risk of malnutrition should be determined using validated tools, such as the Nutritional Risk Screening 2002 (NRS2002), the Malnutrition Universal Screening Tool (MUST), or the Royal Free Hospital-Nutritional Prioritizing Tool (RFH-NPT) (4–6). In the case of a positive risk screening, malnutrition is diagnosed according to GLIM by combining at least one phenotypic criterion (unintentional weight loss, low body mass index (BMI), or reduced muscle mass) with at least one etiological criterion (reduced food intake/malabsorption or disease burden accompanied by some degree of inflammation) (7). However, malnutrition often goes unrecognized due to a lack of awareness, knowledge, clinical protocols and equipment, or because disease-related difficulties complicate diagnostic assessment (8).

Some gastrointestinal diseases may further limit the practical applicability of the GLIM criteria. Patients with chronic diseases of the liver, intestine, and pancreas are often malnourished due to the interrelated functions of these organs in digestion, nutrient absorption, and synthesis of major plasma proteins. Up to 60% of patients with advanced chronic liver disease are malnourished (9, 10). However, overhydration in liver cirrhosis (LC), i.e., edema and ascites, masks weight loss, and distorts BMI and body composition measurements potentially leading to an underestimation of the malnutrition diagnosis. Short bowel syndrome is the result of extensive intestinal loss and the resulting insufficient absorptive capacity for water, electrolytes, and nutrients (11). Malnutrition has been reported in more than 50% of patients with short bowel and intestinal insufficiency (SB/II) (10, 12). However, dehydration, malabsorption, and/or hyperphagia are common in these patients, further complicating nutritional assessment (13). Malnutrition is also common in patients with chronic pancreatitis (CP) due to exocrine and endocrine insufficiency. We recently reported a malnutrition prevalence of 64%, mainly characterized by loss of skeletal muscle mass although this condition becomes apparent only in advanced stages (14). Therefore, an evaluation of the current diagnostic criteria and possibly the implementation of additional criteria is needed for a reliable and clinically applicable assessment of malnutrition in patients with different chronic gastrointestinal diseases.

In this context, state-of-the-art *in silico* data analysis could be helpful for the early detection of malnutrition. The use of Machine Learning (ML) in biomedical research has increased in recent years, not only because of the increasing size and complexity of biomedical data. By fitting predictive models to data and applying feature prioritization and explainability techniques to the fitted model, supervised ML can help to understand the association of various biological and clinical factors (ML features) with diseases/phenotypes (ML labels), thereby deepening the understanding of biomedical data and underlying biological processes (15). In addition, unsupervised ML can identify patterns in unlabeled data sets and therefore may recognize previously undetected clusters in larger data sets such as different patient groups (16). Finally, decision trees are a helpful tool for identifying decision rules based on identified features and cut-off values, allowing the transition of ML-driven decision making into practice (15).

In the present study, we analyzed comprehensive data sets of patients with different chronic gastrointestinal diseases (LC, SB/II, and CP) and two control groups using ML to identify previously unknown and potentially specific features of their malnutrition and to validate the relevance of the GLIM criteria for clinical practice.

## Materials and methods

### Declarations about the data

This study was conducted as part of the collaborative EnErGie project (ESF/14-BM-A55-0007/18). Data were collected between 10/2018 and 09/2021 from the cross-sectional EnErGie study. The study was approved by the institutional review board of the Rostock University Medical Center (A2018-0129) and registered in the Clinical Trials Register (NTC04474743) and the German Clinical Trials Register (DRKS00021124). All patients provided written informed consent. All examinations were performed by trained study personnel.

**Figure 1:**
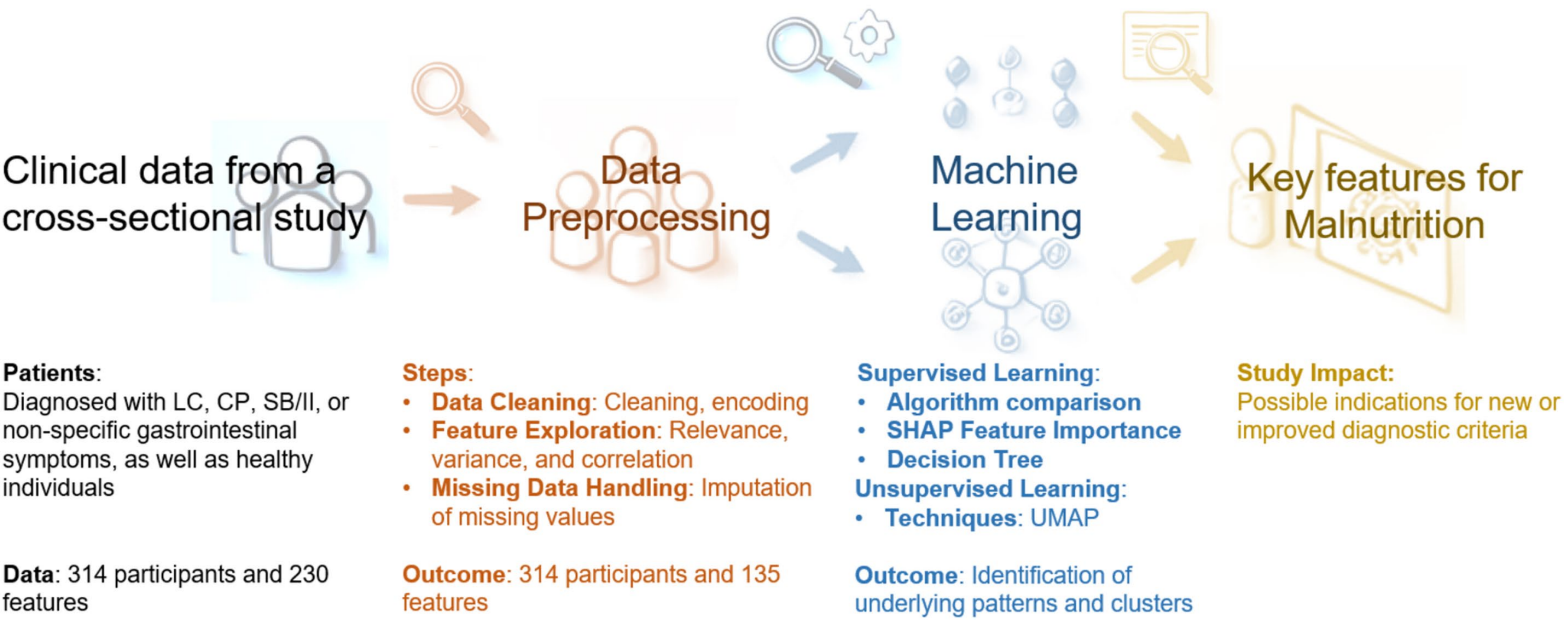
Overview of the data processing. LC, liver cirrhosis; CP, chronic pancreatitis; SB/II, short bowel/intestinal insufficiency; SHAP, SHapley Additive exPlanations; UMAP, uniform manifold approximation and projection.

An overview of the data processing described in detail below is shown in Figure 1.

### Curation of the clinical data set

A total of *n* = 314 subjects were enrolled, including patients with chronic gastrointestinal diseases (LC, CP, SB/II), as well as patients referred for subacute non-specific complaints (control patients (controls)), and healthy controls (HC). All participants were at least 18 years of age. Individuals were excluded from the study if any of the following criteria were met: any parenteral nutrition in the past 6 months, ongoing nutritional intervention (oral nutritional supplements, enteral or parenteral nutrition) for >7 days, trans-jugular intrahepatic portosystemic shunt (TIPS), previous liver transplantation, acute phase of intestinal insufficiency (<28 days after bowel resection), pregnancy/breastfeeding period, pacemaker/implanted defibrillator, malignancy in the past 3 years, and insufficient ability to answer questionnaires.

Patient data were obtained from the medical records and by interview. Anthropometric data, body composition [by bioelectrical impedance analysis (BIA)], muscle strength (by measurement of maximal handgrip strength) and muscle function (by measurement of gait speed), and blood parameters were determined. Disease severity was documented for LC patients (Child-Pugh Score) and CP patients (Chronic Pancreatitis Prognosis Score, COPPS). In addition, questionnaires on diet quality and frequency [“Studie zur Gesundheit Erwachsener in Deutschland (DEGS),” Study of Health in Pomerania (SHIP)], physical activity [International Physical Activity Questionnaire (IPAQ)], and mental health (Hospital Anxiety and Depression Scale (HADS), Fatigue Severity Scale (FSS), de Jong Gierveld and van Tilburg Scale) were applied.

Malnutrition risk was assessed by NRS-2002 or RFH-NPT (the latter in LC patients), and malnutrition was diagnosed using the GLIM algorithm (7). Briefly, the diagnosis of malnutrition requires the presence of at least one phenotypic [unintentional weight loss, low BMI, reduced fat-free mass index (FFMI)] and one etiologic criterion (reduced food intake or malabsorption, disease burden accompanied by some degree of inflammation). The criteria were applied as described previously (10). C-reactive protein (CRP) of >5 mg/l was used as a proxy for inflammation. A total of *n* = 230 variables (hereafter referred to as features) were recorded (Supplementary Table S1).

### Data preprocessing

As reviewed in detail by Fan et al., data preprocessing is necessary to handle irregular, noisy, and missing data in biomedical data sets (17). Initial data cleaning measures included verification, de-duplication, reformatting, and standardization of the data points (Figure 1).

Since most ML models can only handle numerical values, it is necessary to convert categorical features into numerical representations (encoding). For example, binary features with categories no/false and yes/true were encoded as 0 and 1, respectively. Other features with more than two or with more complex categories were encoded using ordinal encoding, where each category is assigned to a unique numerical value.

Further preprocessing included feature exploration and exclusion of features with high number of missing values, redundancy, high variance, and high correlation between the features. Missing values were imputed using the mean or most common value for continuous or categorical features, respectively, across all patients with the same sex and chronic gastrointestinal disease. Finally, *n* = 135 of the original *n* = 230 features were selected for further analyses (see Supplementary Table S1).

### Supervised machine learning

In order to identify the key features that are most important for the diagnosis of malnutrition, we investigated the contribution of the selected features to the classification of non-malnourished versus malnourished. The GLIM diagnosis was used as the label, leaving *n* = 134 features for supervised ML analysis. Several classification algorithms were compared: Adaptive Boosting (AdaBoost), decision trees, the K-Nearest Neighbors algorithm (KNN), Light Gradient Boosting (LGBM), Logistic Regression, the Naive Bayes classifier, Random Forests, Support Vector Machines (SVM), and eXtreme Gradient Boosting (XGBoost) [reviewed in Akpan and Starkey (18) and in Sen et al. (19)]. In each case, the data was randomly divided into a training set (80% of the samples) and a test set (20% of the samples) in a stratified manner, ensuring that the training and test sets had the same ratio of malnourished to non-malnourished patients as the original data set. Additionally, the models were trained in one of the following scenarios: (a) using the complete set of *n* = 134 features or (b) omitting the features used to obtain the GLIM diagnosis: total weight loss (%), BMI, FFMI, reduced food intake, and disease/inflammation. In addition, the features ‘malnutrition risk’ and ‘CRP’, which was used as a supporting proxy to assess disease/inflammation, were omitted because they are associated with the diagnostic features. The second scenario resulted in a total number of *n* = 127 features.

There was a slight imbalance in the data regarding the diagnosis of malnutrition, with an imbalance ratio of about 1:3, i.e., the dataset contained fewer malnourished than non-malnourished participants. Since imbalanced data can affect model performance, this imbalance was addressed by using cost-sensitive learning, if applicable within the algorithm. Hyperparameters, which are used to control the learning process of the algorithm, were optimized using a grid search approach, with a strong emphasis on parameters that counteract overfitting. All models underwent *n* = 100 repetitions of 10-fold stratified cross-validation to avoid overfitting and to evaluate the ability of the model to predict unseen data (20). Model performance was assessed by determining accuracy, precision, sensitivity, specificity, the area under the receiver operating characteristic (ROC AUC), the average precision score, the F1-score, balanced accuracy, and Cohen’s kappa score (21). The last four metrics are particularly useful when there is imbalance in the data. The mean and standard deviation were then calculated for each metric over the 100 × 10-fold cross-validation iterations (*n* = 1,000).

For the best-performing model from each of the two scenarios, SHapley Additive exPlanations (SHAP) were used to assess the extent to which a feature influences the model’s prediction (22). The SHAP approach calculates the contribution of each feature to the model output and thus provides insight into the decision of a model. The average SHAP value for each feature and participant was calculated over the 100 × 10-fold cross-validation iterations (adapted from (23)). Based on the SHAP values, the 10 most important features for identifying malnourished patients were identified.

All supervised machine learning methods (including decision trees, see below) were performed in Python (version 3.9.16) using scikit-learn (version 1.0.1).

### Unsupervised machine learning

Dimension reduction and visualization of the underlying patterns within the patient data was performed using Uniform Approximation and Projection (UMAP) (24). Due to the diversity of feature types within the data set, the Feature-Type Distributed Clustering (FDC) approach was applied as previously described by our group (25). The consideration of diverse feature types in combination with the conventional UMAP algorithm can lead to more effective results when investigating underlying patterns or patient clusters from patient data (25).

Accordingly, the UMAP algorithm was applied to the ordinal, nominal, and continuous features separately using the Canberra, Hamming, and Euclidean distance metrics, respectively. For all other parameters, default values were found to be optimal. Then, both dimensions of each of the ordinal and continuous projections and one of the nominal dimensions were integrated to reduce the high-dimensional data to an intermediate 5-dimensional embedding. This 5-dimensional embedding was further processed with conventional UMAP (using default UMAP parameters) to obtain the final 2D UMAP projection.

### Decision trees

The 10 selected key features from the best performing model using all features were used to train decision trees to investigate the explanatory power of the ML models. As described above for supervised ML, the data was shuffled and stratified into training data (80%) and test data (20%). The hyperparameters were optimized using a grid search approach. The tree was trained to a maximum depth of three. After training the model on the training data, the performance of the tree was evaluated on the test data using the same metrics as in the initial approach to compare the different algorithms. From five randomly generated decision trees, the best performing model was selected to determine and visualize its decision rules, including the respective features and cut-off values, when classifying the data.

### Statistical analyses

Statistical analyses were performed using IBM SPSS Statistics (version 28, Ehningen, Germany) to compare the distribution of sex, age, and malnutrition diagnosis between patients with different chronic gastrointestinal diseases and controls in order to characterize the different patient and control cohorts. In addition, statistical tests were performed to compare features within clusters identified by ML as well as to compare features in patients with and without malnutrition diagnosis. After testing for normal distribution, the Mann–Whitney-U test or Student’s t-test was used for interval scaled variables. Nominal and ordinal scaled variables were tested using Pearson chi-squared test with Bonferroni adjustment. Data are presented as absolute (n) and relative (%) values, mean ± standard deviation (SD; normally distributed data) or median and interquartile range (IQR, non-normally distributed data). Statistical significance was considered when *p* < 0.05.

## Results and discussion

Disease-related malnutrition is a common complication in the hospital setting, with a particularly high prevalence in chronic gastrointestinal diseases (1). Despite the availability of diagnostic criteria, malnutrition often remains unrecognized or underestimated, and individual criteria are limited in their practical applicability in several gastrointestinal diseases, such as LC. Machine Learning (ML) is a contemporary approach that can be used to evaluate current diagnostic criteria, to identify additional features and a core data set for malnutrition diagnosis and potentially provide insights into the underlying mechanisms of malnutrition. The use of ML to aid diagnosis has already been evaluated in elderly patients with malnutrition, highlighting the potential of these technologies to improve diagnostic accuracy (26). However, in the context of ML, there are currently only a few studies investigating the interplay between malnutrition and chronic gastrointestinal diseases such as liver cirrhosis.

Furthermore, the GLIM criteria were developed based on expert consensus (7), and the use of ML algorithms can be used as a more unbiased strategy to develop additional or optimize the current diagnostic criteria. Therefore, the aim of this study was to analyze a comprehensive data set using ML to characterize both general and specific features of malnourished patients with different chronic gastrointestinal diseases and to validate the relevance of the GLIM criteria for clinical practice.

### Demographic data

The demographic data of the different groups and the prevalence of malnutrition are summarized in Table 1. Most of the data sets were derived from HC, and patients with LC or CP, while SB/II and control patients comprised smaller groups. Age was comparable between control and patient groups. The percentage of women was >50% in the HC and control patients as well as in SB/II. In contrast and as expected, fewer patients were female in the LC (33%) and CP (23%) groups. While none of the HC was malnourished, 34% of the control patients were diagnosed with malnutrition. However, this was not unexpected in control patients with subacute non-specific gastrointestinal complaints. Malnutrition was more prevalent among LC patients (>60%) compared to the other groups. The data set included patients with varying degrees of the disease severity (LC: Child-Pugh Score A 19.3%, B 41.0%, C 39.7%; CP: COPSS A 33.3%, B 49.3%, C 17.4%, data not shown). The prevalence of malnutrition was found to increase with progressive disease. In LC patients, malnutrition was diagnosed in 40.0% with Child-Pugh Score A, in 68.8% with Child-Pugh Score B and in 61.3% with Child-Pugh Score C. In CP patients, malnutrition was diagnosed in 13.0% with COPPS A, in 44.1% with COPPS B and in 83.3% with COPPS C (data not shown). These findings indicate that malnutrition is a common complication in both (sub-)acute and chronic gastrointestinal diseases and increases with disease progression.

**Table 1:** Overview about the implemented cohorts of healthy controls (HC), control patients (controls) and patients with liver cirrhosis (LC), chronic pancreatitis (CP) as well as short bowel/intestinal insufficiency (SB/II).

	Total (*n* = 314, 100.0%)	HC (*n* = 91, 29.0%)	Controls (*n* = 47, 15.0%)	LC (*n* = 78, 24.8%)	CP (*n* = 71, 22.6%)	SB/II (*n* = 27, 8.6%)	*p* value
Sex, female [*n* (%)]	132 (42.0)	47 (51.6)	26 (55.3)	26 (33.3)	17 (23.9)*	16 (59.3)	**<0.001** ^**b**^
Age [years]	58 (16)	58 (16)	54 (27)	58 (11)	58 (18)	60 (22)	0.109^a^
Malnutrition (diagnosed by GLIM) [*n* (%)]	104 (33.1)	0 (0)*	16 (34.0)	47 (60.3)*	30 (42.3)	11 (40.7)	**<0.001** ^**b**^

Data are shown as absolute (*n*) and relative values (%) as well as median (IQR) (age).

^a Kruskal-Wallis test.^

^b^ Pearson chi-squared test with Bonferroni adjustment (* indicates statistical significance).

Bold values indicate statistical significance (*p* < 0.05).

### Supervised classification of malnutrition

The results of the supervised ML analyses are described and discussed together in the following sections.

#### High classification performance after inclusion of all clinical features

We performed a classification analysis of the malnourished versus the non-malnourished patients and controls using all 134 selected features. Nine different classification models were used for this purpose and a number of different performance metrics were obtained. The results of the analysis are summarized in Table 2 (sensitivity, specificity, and F1-score) and in Supplementary Table S2 (additional performance metrics).

**Table 2:** Average performance of the trained models classifying malnutrition using all (*n* = 134) features.

	Decision tree	KNN	SVM	Logistic regression	Naive Bayes	Random Forest	AdaBoost	LGBM	XGBoost
Sensitivity	0.875 ± 0.107	0.491 ± 0.152	0.807 ± 0.123	**0.903 ± 0.096**	0.696 ± 0.140	0.849 ± 0.107	0.863 ± 0.109	0.888 ± 0.101	0.849 ± 0.112
Specificity	0.909 ± 0.070	**0.954 ± 0.046**	0.852 ± 0.077	0.843 ± 0.078	0.841 ± 0.083	0.907 ± 0.065	0.941 ± 0.055	0.935 ± 0.054	0.935 ± 0.053
F1-score	0.85 ± 0.085	0.608 ± 0.139	0.766 ± 0.09	0.815 ± 0.076	0.688 ± 0.110	0.833 ± 0.082	0.870 ± 0.079	**0.879 ± 0.075**	0.856 ± 0.078

Performance was evaluated for 100 × 10-fold cross-validation iterations (*n* = 1,000). The highest value for each performance metric across all algorithms is highlighted. Data are shown as mean ± standard deviation. KNN, K-nearest neighbors; SVM, support vector machine; AdaBoost, adaptive boosting; LGBM, light gradient boosting machine; XGBoost, eXtreme gradient boosting. Bold values indicate statistical significance (*p* < 0.05).

The LGBM classifier performed best in terms of F1-score, which is a widely used performance metric for imbalanced data sets that combines precision and sensitivity (Table 2). In addition, LGBM had the best performance in terms of accuracy, ROC AUC, balanced accuracy, and Cohen’s kappa score (Supplementary Table S2). KNN, a simpler classification model, performed best in terms of specificity, and Logistic Regression performed best in terms of sensitivity. Given that at least one classifier can achieve a score of more than 80% for both the F1 and the Cohen’s kappa score, we conclude that the imbalance in the data does not have a negative effect on the classifiers. This is also supported by the high values of the average precision score and the balanced accuracy (Supplementary Table S2). Taken together, the ensemble models such as Random Forests, Adaboost, LGBM, and XGBoost perform better than the simpler classifiers. Since the LGBM had the best performance for classifying malnourished patients, we further investigated which features were mainly used by this model.

#### Reduced classification performance after removal of the GLIM criteria

The GLIM criteria were used for operational diagnosis of malnutrition. Therefore, it is clear that these criteria are associated with the state of malnutrition. The search for additional features that are independently associated with the diagnosis of malnutrition may identify biomarkers for malnutrition or provide insight into the mechanisms of malnutrition. Therefore, a classification analysis was performed without the GLIM criteria and associated features.

The performance metrics are shown in Table 3 (sensitivity, specificity, and F1-score) and Supplementary Table S3 (additional performance metrics). The Random Forest model produces the highest F1-score, accuracy, ROC AUC, average precision score, balanced accuracy, and Cohen’s kappa score. The KNN model, on the other hand, produces the highest measures of precision and specificity, while the decision tree produces the best sensitivity. Again, the average precision score, F1-score, balanced accuracy, and Cohen’s kappa score of the best performing classification models are quite high, indicating that the imbalance present in the data does not affect the classification. The Random Forest model was selected for further interpretive analyses because it was the best performing model when GLIM features were omitted from the calculations.

**Table 3:** Average performance of the trained models classifying malnutrition leaving out GLIM diagnosis criteria (total weight loss, BMI, FFMI, CRP, reduced food intake, chronic disease/inflammation, malnutrition risk; *n* = 127).

	Decision Tree	KNN	SVM	Logistic regression	Naive Bayes	Random Forest	AdaBoost	LGBM	XGBoost
Sensitivity	**0.869 ± 0.119**	0.390 ± 0.144	0.709 ± 0.135	0.770 ± 0.124	0.603 ± 0.147	0.721 ± 0.132	0.679 ± 0.144	0.731 ± 0.136	0.667 ± 0.146
Specificity	0.677 ± 0.111	**0.926 ± 0.056**	0.818 ± 0.082	0.732 ± 0.095	0.834 ± 0.084	0.862 ± 0.074	0.853 ± 0.078	0.839 ± 0.080	0.860 ± 0.076
F1-score	0.690 ± 0.082	0.496 ± 0.143	0.681 ± 0.103	0.667 ± 0.092	0.619 ± 0.120	**0.720 ± 0.102**	0.684 ± 0.109	0.709 ± 0.102	0.680 ± 0.111

Performance was evaluated for 100 × 10-fold cross-validation iterations (*n* = 1,000). The highest value for each performance metric across all algorithms is highlighted. Data are shown as mean ± standard deviation. KNN, K-nearest neighbors; SVM, support vector machine; AdaBoost, adaptive boosting; LGBM, light gradient boosting machine; XGBoost, eXtreme gradient boosting. Bold values indicate the highest performance value.

As expected, the classification performance of the models decreased with the exclusion of the GLIM criteria highlighting their importance in the diagnosis of malnutrition. Compared to the LGBM as the best performing model including the GLIM criteria, the Random Forest model lost 19% sensitivity (0.888 ± 0.101 vs. 0.721 ± 0.132), and 8% specificity (0.935 ± 0.054 vs. 0.862 ± 0.074), while the F1-score decreased by 10% (0.879 ± 0.075 vs. 0.792 ± 0.075).

#### Supervised machine learning identified key features associated with malnutrition

**Figure 2:**
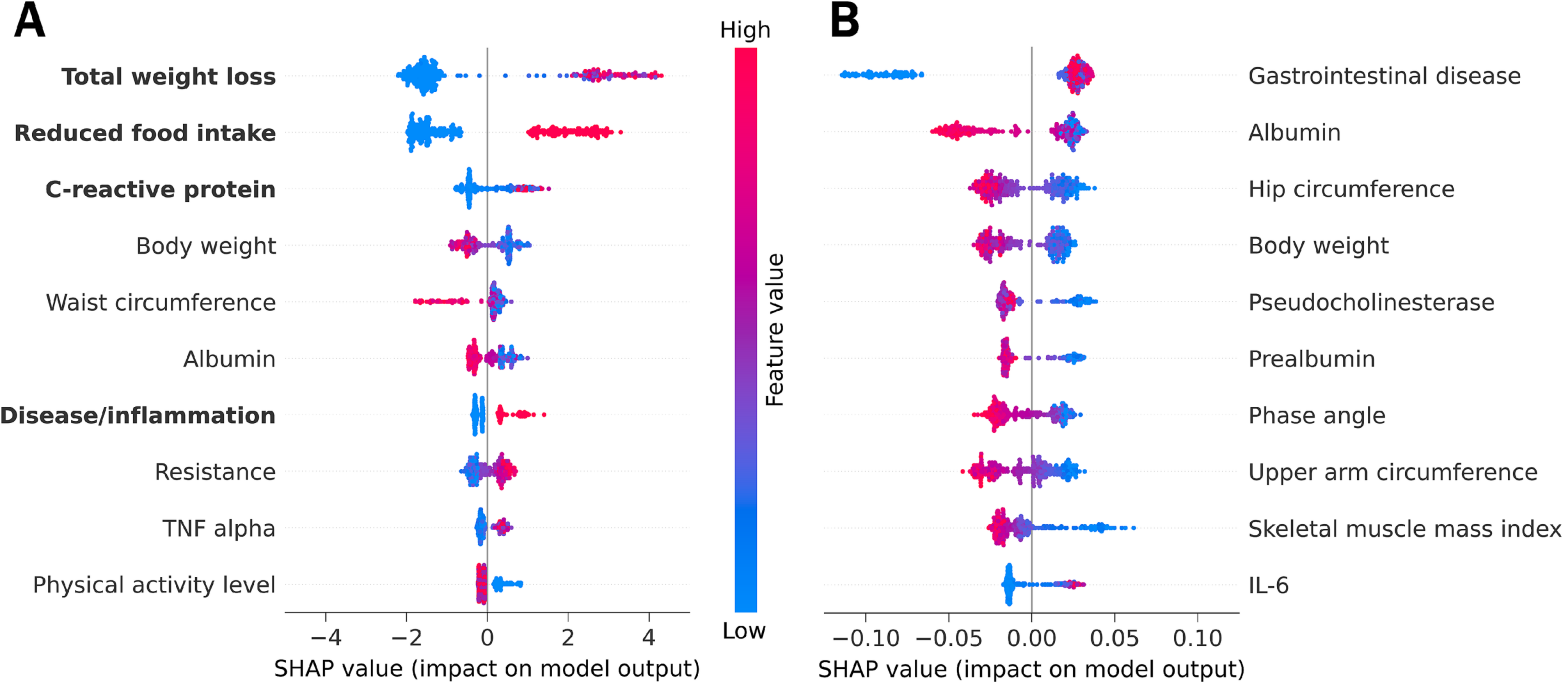
SHAP feature importance summary plot from **(A)** the LGBM using all features including GLIM diagnostic criteria (marked in bold) or from **(B)** the Random Forest model omitting the GLIM malnutrition diagnosis criteria and the associated features malnutrition risk and C-reactive protein. Each dot represents the average SHAP feature importance value for one patient regarding the respective feature. The color of the dot represents the feature expression value (red - high, blue - low). Positive SHAP values indicate classification of malnourished patients, while negative SHAP values indicate classification of non-malnourished individuals. An alignment to the right therefore indicates higher importance of a feature for malnutrition classification. IL-6, interleukin 6; TNF, tumor necrosis factor.

Both supervised ML approaches (with and without GLIM criteria) identified a list of features that contribute to the classification of malnourished versus non-malnourished. SHAP values which reflect the impact of the features on the model output, were used to prioritize the features in terms of their relevance for diagnosing malnutrition. Figure 2 shows the ten most important features for the approach including the GLIM criteria (A) or excluding the GLIM criteria, as well as malnutrition risk and CRP as a proxy for inflammation (B). For each participant and feature, the average SHAP value is shown as a single point. Overall feature importance was calculated as the mean of the absolute SHAP values across all participants for each feature.

##### Best model using the GLIM criteria

The LGBM was used as the best performing model considering all features (Figure 2A, see also Table 2). The main results using this approach are listed below and discussed in the next section.

According to the SHAP values, the GLIM phenotypic criterion of unintentional total weight loss was the most important feature for classifying malnutrition. Total weight loss was more important for classifying malnourished than non-malnourished patients/controls (alignment more to the right). High weight loss (indicated by the red color) increases the model output for positive malnutrition classification (positive SHAP values).

The other phenotypic criteria (reduced FFMI, position 17; and low BMI, position 35; Supplementary Table S4) were considered less important for malnutrition diagnosis using the supervised ML approach. The most likely reason for this is the high proportion of overhydrated LC patients, which masks the body weight and alters body composition.

The etiologic criteria (reduced food intake, disease burden accompanied by inflammation, and CRP as a supportive proxy for this criterion) were other key features of the malnutrition diagnosis. Reduced food intake and disease burden as well as high CRP levels are positively associated with the diagnosis of malnutrition. The assessment of reduced food intake provided a particularly reliable classification of malnourished and non-malnourished patients and controls. Another inflammatory marker, tumor necrosis factor (TNF)-alpha is among the 10 features with the highest SHAP values.

In addition, the anthropometric measures of body weight and waist circumference were key features for malnutrition diagnosis, with lower feature values increasing model output toward malnutrition diagnosis. However, high waist circumference was more important for classifying non-malnourished patients and controls.

Serum albumin, often used as clinical laboratory marker for malnutrition but now considered as a marker of inflammation and disease severity (27), was also identified as an important feature for the model output.

Resistance (the ohmic resistance in alternative current, a raw value measured by BIA) and the physical activity level as a functional parameter were also among the 10 most important features predictive of malnutrition diagnosis using the LGBM.

##### Best model without GLIM criteria

We also applied Random Forest as the best performing model omitting the diagnostic GLIM criteria and malnutrition risk, to investigate which features have an additional impact and are still able to predict malnutrition when the GLIM criteria are not considered for classification (Figure 2B, see also Table 3). The lower numerical SHAP values were expected and are not solely related to the omission of the criteria, as Random Forest yields lower values compared to LGBM. The ten key features of the malnutrition diagnosis generated by this approach are listed below.

An underlying chronic gastrointestinal disease was found to be the most important feature for diagnosing malnutrition when malnutrition risk and the diagnostic criteria are excluded. The absence of an underlying disease was more important for the classification of non-malnourished patients and controls, while the presence of a disease state was weakly associated with malnutrition.

The laboratory parameters albumin, pseudocholinesterase, and prealbumin were also identified as key features. Lower values had a greater impact on the model output toward the diagnosis of malnutrition.

The anthropometric measures of hip circumference, body weight, and upper arm circumference had a high impact on the model output. Lower feature values had higher SHAP values associated with a positive diagnosis of malnutrition.

Phase angle and SMMI are features of body composition measurements. According to our model, low feature values are associated with the diagnosis of malnutrition.

Furthermore, inflammation was considered important for malnutrition classification with the pro-inflammatory cytokines Interleukin-6 (IL-6) and TNF-alpha (position 11, see Supplementary Table S5) being key features identified by ML. Low levels were associated with both malnourished and non-malnourished patients, making classification unreliable. However, high levels of inflammatory cytokines were associated with a positive diagnosis of malnutrition.

#### Biological significance of the ML-identified key features

To assess the biological significance of the above listed findings, we compared the numerical values of the ML-identified key features between non-malnourished and malnourished patients (Table 4). All features are highly significantly different between the two groups, highlighting their relevance to malnutrition and confirming the functionality of the presented ML approach. Despite the biologically important differences in the numerical values, there are overlapping ranges which means that not all of these parameters are suitable as biomarkers. However, the inclusion of additional features to the established GLIM criteria could improve the diagnostic accuracy in chronic gastrointestinal diseases. Furthermore, the key features identified may be indicators of mechanisms or different manifestations of malnutrition.

**Table 4:** Comparison of the ML-identified key features of malnutrition diagnosis between non-malnourished and malnourished patients.

	Non-malnourished (*n* = 210)	Malnourished (*n* = 104)	*p* value
Total weight loss, %	0.0 (0.0)	11.8 (15.1)	**<0.001** ^**a**^
Reduced food intake, yes (%)	42 (20.0%)	82 (78.9%)	**<0.001** ^**c**^
C-reactive protein, mg/l	2.4 (3.8)	9.9 (31.7)	**<0.001** ^**a**^
Body weight, kg	80.8 (22.9)	70.5 (17.3)	**<0.001** ^**a**^
Waist circumference, cm	95.77 ± 16.03	91.76 ± 15.49	**0.036** ^**b**^
Albumin, g/l	43.1 (7.8)	35.0 (12.0)	**<0.001** ^**a**^
Disease/inflammation, yes (%)	50 (23.8%)	68 (65.4%)	**<0.001** ^**c**^
Resistance, *Ω*	563.37 ± 90.66	617.71 ± 117.10	**<0.001** ^**b**^
TNF-alpha, pg./ml	5.5 (3.7)	9.5 (8.4)	**<0.001** ^**a**^
Physical activity level, low (%) / moderate (%) / high (%)	48 (22.9%) / 78 (37.1%) / 84 (40.0%)	53 (51.0%) / 26 (25.0%) / 24 (23.1%)	**<0.001** ^**c**^
Gastrointestinal disease, yes (%)	88 (41.9%)	88 (84.6%)	**<0.001** ^**c**^
Hip circumference, cm	102.1 (12.7)	95.0 (11.3)	**<0.001** ^**a**^
Pseudocholinesterase, kU/l	8.00 (3.35)	4.84 (7.81)	**<0.001** ^**a**^
Prealbumin, g/l	0.23 (0.09)	0.12 (0.20)	**<0.001** ^**a**^
Phase angle, °	5.0 (1.0)	4.2 (1.3)	**<0.001** ^**a**^
Upper arm circumference, cm	30.4 (5.6)	26.6 (5.6)	**<0.001** ^**a**^
Skeletal muscle mass index, kg/m^2^	8.58 ± 1.59	7.17 ± 2.13	**<0.001** ^**b**^
IL-6, pg/ml	2.32 (3.50)	13.30 (40.0)	**<0.001** ^**a**^

Data are shown as absolute (*n*) and relative values (%), mean ± standard deviation or median (IQR).

^a Mann–Whitney-U test.^

^b Student’s T-test.^

^c Pearson chi-squared test.^

Bold values indicate statistical significance (*p* < 0.05).

##### Significance of the GLIM criteria

Summarizing the results of the supervised ML analysis (Figure 2A), the LGBM identified several GLIM criteria as key features for diagnosing malnutrition from an extensive set of clinical features in a total cohort of patients with chronic gastrointestinal diseases. Among these, total weight loss was the most important feature for classifying malnutrition. Although weight loss may be masked by fluid overload in patients with LC, this criterion had high diagnostic accuracy and was identified as an important driver in the diagnosis of malnutrition by Bannert et al. (10). Weight loss in cirrhosis is related to etiology. Anastácio et al. found that patients with alcoholic liver disease had higher weight loss compared to other etiologies of chronic liver disease (28). In this study, 74% of LC patients (representing 25% of the total cohort) had alcoholic liver disease, which may contribute to the high diagnostic accuracy and model output. However, even a simple history of unintentional and progressive weight loss is considered as a valid predictor of the clinical outcome (29).

Both etiologic GLIM criteria “reduced food intake/malabsorption” and “chronic disease accompanied by inflammation” were other key features, highlighting their importance in classifying malnutrition in chronic gastrointestinal diseases. In our previous study, these diagnostic criteria were most frequently found in this cohort of patients (10). In this context, the inflammatory marker CRP was one of the top features. TNF-alpha and IL-6 were also found to be key features in classifying malnutrition, making them further suitable proxies for malnutrition-related inflammation, but they are often not available in daily clinical practice. According to a Canadian comparative study assessing malnutrition at hospital admission, the best combination of the GLIM diagnostic criteria with fair validity for diagnosing malnutrition was weight loss with either high CRP or low food intake (30). This is consistent with our top three most important features using a ML approach in patients with chronic gastrointestinal disease.

Our results underline the reasonable applicability of the GLIM criteria in patients suffering from different gastrointestinal diseases. It should be emphasized that our less biased ML approach thus confirms the expert consensus and contributes to the validation of the relevance of the consensus criteria for clinical practice, as suggested by the GLIM Committee (7).

##### Significance of other key features

In addition to the GLIM criteria, other key features for classifying malnutrition in patients with chronic gastrointestinal diseases were identified by both supervised ML approaches. Consistent with the reported high prevalence in gastroenterology (1), an underlying chronic gastrointestinal disease was the most important feature for diagnosing malnutrition in our study using a Random Forest model after omitting the GLIM criteria. This highlights the importance of malnutrition screening in patients with a significant gastrointestinal disease (7, 29).

In addition, anthropometric and body composition parameters are of great importance. Reduced hip circumference allowed a reasonably reliable classification of malnourished patients identified by Random Forest. To date, only one study has identified a hip circumference < 88 cm as a risk factor for malnutrition in type 2 diabetic patients with pulmonary tuberculosis (31). In our cohort of 314 participants, hip circumference was significantly decreased in malnourished compared to non-malnourished patients. Therefore, this may be an additional helpful marker in the diagnosis of malnutrition, especially in patients with ascites where other anthropometric and body composition parameters may be masked.

Furthermore, a reduced phase angle, higher resistance value, and lower skeletal muscle mass index indicated a diagnosis of malnutrition using the Random Forest model after excluding the GLIM criteria. Phase angle is a measured parameter of bioelectrical impedance analysis and an index of cell membrane integrity and vitality. It has been shown to be a reasonable indicator of mortality in many clinical situations and is associated with the nutritional status, prognosis, stage, and severity of digestive and liver diseases (32–34). However, the use of the phase angle as a biomarker of malnutrition in gastrointestinal diseases is limited because it is influenced by age, sex, BMI, and inflammation, which affects fluid distribution; therefore, assessment of hydration should be included (35). Skeletal muscle mass index measured by bioelectrical impedance analysis is an indicator for the GLIM criterion reduced muscle mass. When methods such as BIA, CT or MRI are not available, arm muscle circumference can be used as an alternative measure (7). This is also reflected in our results, which identified upper arm circumference as another key feature for classifying malnutrition.

Finally, among the laboratory values, plasma albumin, prealbumin, and pseudocholinesterase, were important features for the model output. Lower values of these parameters in LC patients, who make up the majority of the patients recruited in this study, reflect reduced liver function and thus the disease state. Low albumin concentrations have been shown to correlate with increased medical complications. However, prolonged protein-calorie restriction was associated with a reduction in body weight but little change in plasma albumin concentrations (29). Albumin (as well as prealbumin and pseudocholinesterase) levels further decrease during acute inflammation. In addition, human albumin is administered in conjunction with therapeutic paracentesis or hepatorenal syndrome in LC. Although this was not the case in the present study, the administration of albumin may influence albumin levels, potentially introducing bias and obscuring the presence of malnutrition. Therefore, albumin should not be considered as a standalone marker of malnutrition, but it may be useful to consider albumin in conjunction with other factors.

It should be noted that some of the key features identified may be the cause and others may be the result of malnutrition. On the one hand, the reduction of anthropometric parameters such as hip and upper arm circumferences will result from reduced energy intake and low physical activity and can therefore be used to detect pre-existing malnutrition in the patients. On the other hand, for example inflammation-related changes in the laboratory markers may suggest the underlying mechanisms of malnutrition. This aspect could be addressed in future research.

In conclusion, we found that weight loss, reduced food intake, inflammatory markers, hip and upper arm circumference, SMMI and phase angle, as well as several laboratory parameters (albumin, prealbumin, pseudocholinesterase) had a particularly high impact on the classification of malnutrition in a cohort of patients with different gastrointestinal diseases. Early detection and appropriate treatment of malnutrition is of great importance to prevent adverse outcomes. Furthermore, predictive models using longitudinal data have shown promise in predicting malnutrition, highlighting the importance of continuous patient monitoring and facilitating early nutritional intervention (36). In two recent studies, ML was used to test different combinations of GLIM criteria for their performance in malnutrition diagnosis and prognosis in LC patients awaiting liver transplantation (37, 38). Mid-arm muscle circumference (reflecting the phenotypic GLIM criterion of reduced muscle mass) and liver disease parameters (used as etiologic criteria) were associated with the diagnosis of malnutrition and were also predictors of 1-year mortality (38). In this context, it will be interesting to further analyze our model in relation to the prognosis of (malnourished) patients with chronic gastrointestinal diseases. Image-based models predicting the nutritional risk from facial feature recognition have opened new avenues as non-invasive diagnostics (39). Taken together, these results and developments collectively represent a remarkable application scenario for ML in the diagnosis and management of malnutrition.

### Unsupervised analysis reveals clustering of malnourished cirrhosis patients

**Figure 3:**
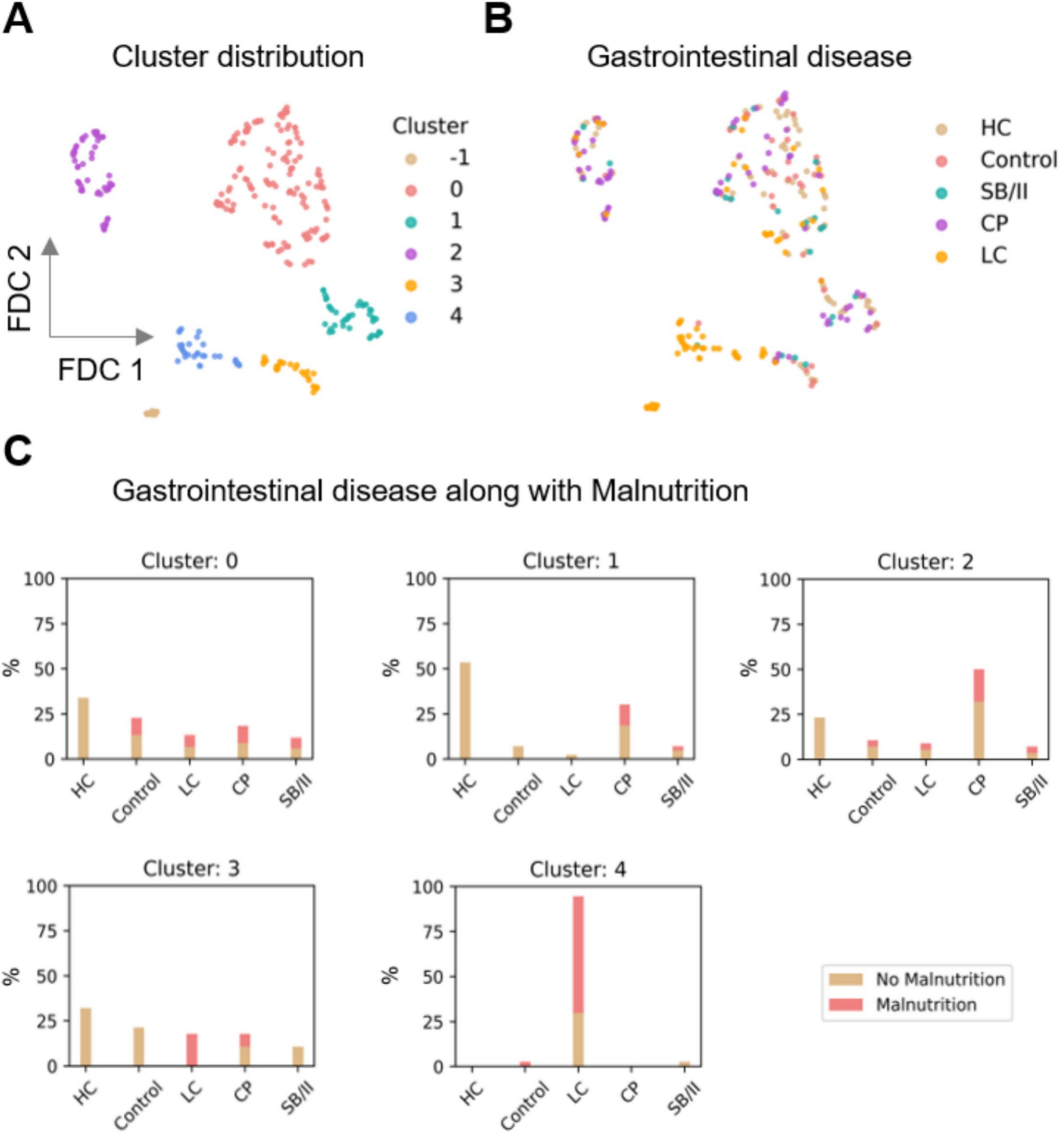
Distribution of the clusters obtained by UMAP supported Feature Distributed Clustering (FDC). **(A)** Five clusters (0–4) were found; moreover 14 data points were denoted as noise (labeled as cluster -1). **(B,C)** Distribution of gastrointestinal diseases along with malnutrition in the clusters. It was observed that cluster 4 is mostly comprised of LC patients and also has the highest proportion of malnourished individuals. UMAP, uniform approximation and projection; HC, healthy controls; SB/II, short bowel/intestinal insufficiency; CP, chronic pancreatitis; LC, liver cirrhosis.

**Figure 4:**
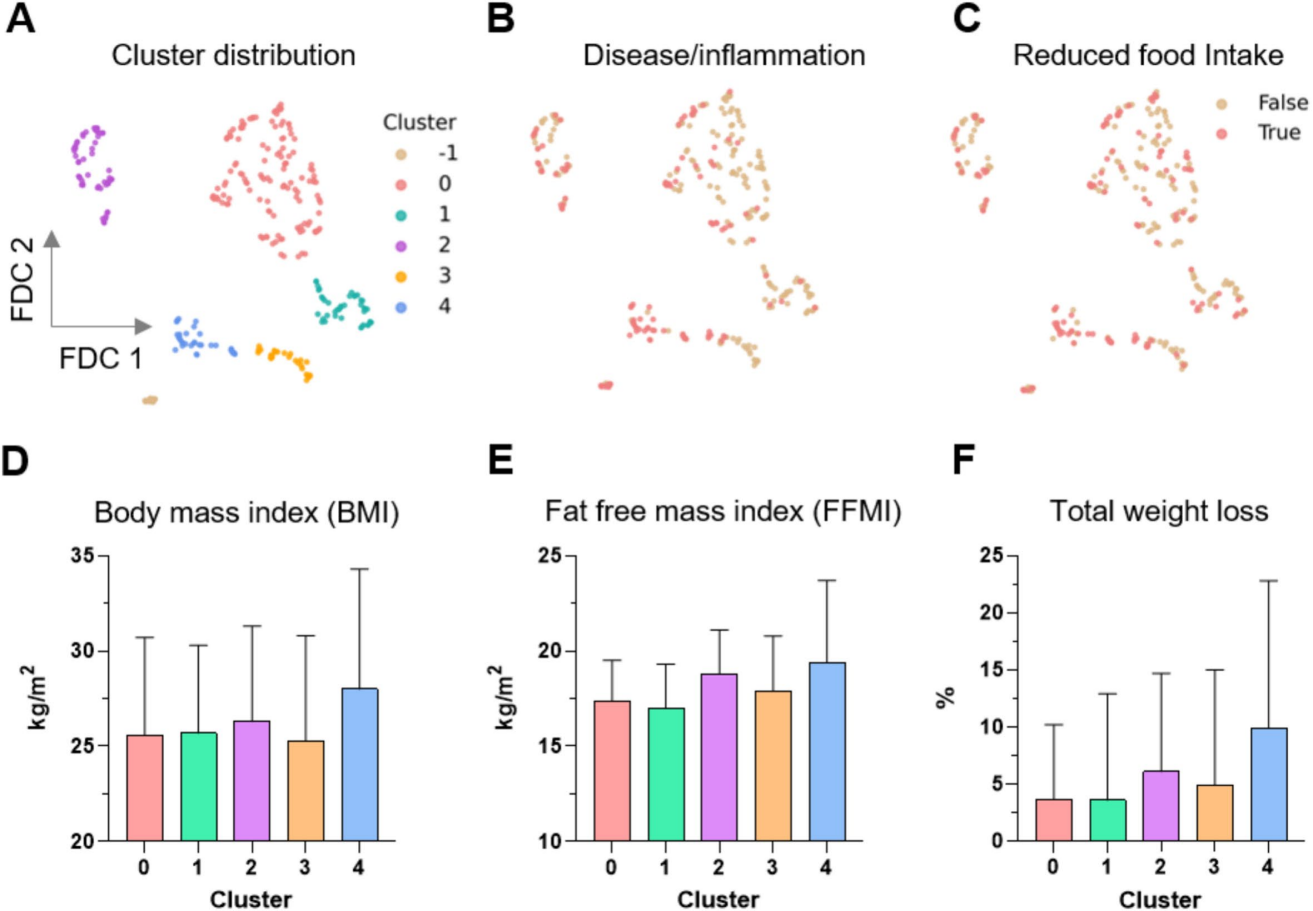
Distribution of the clusters obtained by UMAP supported Feature Distributed Clustering (FDC). **(A)** Five clusters (0–4) were found; moreover 14 data points were denoted as noise (labeled as cluster -1). **(B–F)** Distribution of the distinct GLIM criteria in the clusters. UMAP, uniform approximation and projection; LC, liver cirrhosis; BMI, body mass index; FFMI, fat-free mass index.

The UMAP dimension reduction tool is an unsupervised ML approach that identifies and visualizes the data underlying structures in high-dimensional data. Using this exploratory approach, we identified clusters of patients and controls based on the similarities in their measurements and analyzed the enrichment of the GLIM criteria in these clusters (Figures 3, 4).

UMAP revealed five clusters in the data, labeled clusters 0, 1, 2, 3, and 4, each consisting of 136, 43, 56, 28, and 37 data points, respectively (Figure 3A). A total of 14 data points were labeled as noise by the Density-Based Spatial Clustering of Applications with Noise (DBSCAN) algorithm (labeled cluster -1 in Figures 3A, 4A). The distribution of the control and patient cohorts among the clusters (Figure 3B) showed that cluster 4 consisted mainly of LC patients and cluster 1 had a high proportion of CP patients and HC, while the other clusters consisted of a heterogeneous mixture of patients with the indicated gastrointestinal diseases and controls. In addition, cluster 4 had the highest proportion of malnourished patients and thus consisted mainly of malnourished LC patients (Figure 3C). Beyond that, malnourished and non-malnourished patients were distributed among the other clusters. We expected from our data set of different and complex features related to the nutritional status that malnourished patients (and controls) would cluster in a separate group compared to well-nourished individuals. However, the five clusters revealed could also indicate that there are more (here in our cohort five) nutritional states. In such a scenario, where the classification into malnourished and non-malnourished does not correspond to the nutritional states identified by individual clusters, this may indicate why the diagnosis of malnutrition is so difficult to define and detect. A deeper analysis involving more patients with a wider variety of diseases and repeated analyses over time in the presence (patients) or absence (HC) of disease is needed to address this.

We further investigated the differential distribution of features among these clusters. We mainly focused on the distribution of the GLIM criteria because our previous results from the supervised ML approach showed that several GLIM criteria serve as key features for diagnosing malnutrition in patients with different gastrointestinal diseases (Figure 4). While the False and True categories of both binary features, disease/inflammation and reduced food intake (Figures 4B,C), are widely distributed in the clusters 0 to 3, cluster 4 is more dominated by positive values (True) for both features. Figures 4D–F shows the distribution of BMI, FFMI, and total weight loss across all clusters.

Despite high standard deviations, ANOVA testing revealed differences between the clusters for all GLIM features except BMI (Table 5). The mean values for FFMI, total weight loss and BMI, as well as the percentage of individuals with reduced food intake and disease/inflammation were higher in cluster 4 compared to the other clusters. This was expected since this cluster consisted mainly of LC patients who often suffer from overhydration, which leads to changes in body composition and limits the reliability of BIA measurement.

**Table 5:** The relative values (%) and mean ± SD of the individual GLIM criteria in the five clusters (0–4) detected by the unsupervised analysis. Cluster 4 shows different patterns compared to the others. ANOVA was used for statistical testing.

	Cluster 0	Cluster 1	Cluster 2	Cluster 3	Cluster 4	*p*-value
BMI, kg/m^2^ (Mean ± SD)	25.6 ± 5.1	25.7 ± 4.6	26.3 ± 5.0	25.3 ± 5.5	28.0 ± 6.3	0.1455
FFMI, kg/m^2^ (Mean ± SD)	17.4 ± 2.1	17.0 ± 2.3	18.8 ± 2.3	17.9 ± 2.9	19.4 ± 4.3	**0.0003**
Total weight loss, % (Mean ± SD)	3.7 ± 6.5	3.6 ± 9.3	6.1 ± 8.6	4.9 ± 10.1	9.9 ± 12.9	**0.0026**
Reduced Food Intake (True, %)	36.0%	25.5%	35.7%	39.2%	81.0%	**<0.0001**
Disease/inflammation (True, %)	27.9%	11.6%	41.0%	25.0%	89.1%	**<0.0001**

BMI, body mass index; FFMI, fat-free mass index. Bold values indicate statistical significance (*p* < 0.05).

Because cluster 4 had the sharpest profile and was clearly different from the others, clusters 0 to 3 were combined for further statistical testing and compared with cluster 4. Cluster 4, which is dominated by (malnourished) LC patients (Figure 3), differed highly significantly in many features: GLIM criteria were higher in cluster 4 (see also Table 5), as was the diagnosis of malnutrition (and also the risk of malnutrition). In addition, several features were found to be different including laboratory parameters, lower physical activity level, reduced skeletal muscle mass, grip strength and gait speed, higher prevalence of edema and ascites associated with increased waist circumference, lower intake of several macro-and micronutrients, presumably due to reduced food intake, and more frequent psychosocial symptoms (anxiety, depression, fatigue and loneliness) (data not shown). This may indicate that the pathogenesis of malnourished LC patients differs from that of malnourished patients with other chronic gastrointestinal diseases and from that of non-malnourished LC patients. Previously, we found that individual GLIM criteria act as specific drivers for the diagnosis of malnutrition in different chronic gastrointestinal diseases, indicating different underlying mechanisms or manifestations of malnutrition (10).

### Interpretable decision making using decision trees

With the intention to translating our *in silico* results into clinical applicability, we generated decision trees to define decision rules and cut-off values for the diagnosis of malnutrition. Yin et al. used tree-based methods to visualize and validate decision tools for identifying malnutrition in cancer patients in a retrospective study, demonstrating the overall utility of such approaches in clinical diagnostics (40).

**Figure 5:**
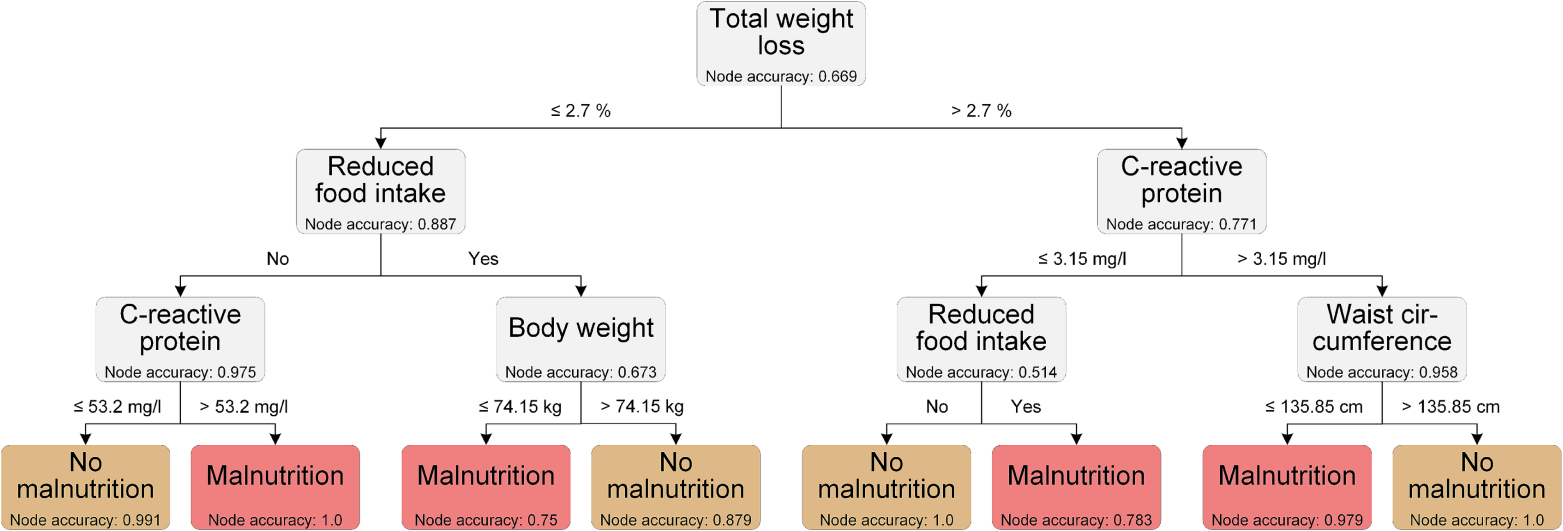
Exemplary decision tree using the top 10 features from LGBM using all features.

The LGBM was the best performing model when considering all the features, so we used the top ten features from the LGBM classification to generate the decision trees. The decision tree with the best performance (overall accuracy 0.968) is shown in Figure 5. This example decision tree includes a phenotypic (total weight loss) and an etiologic GLIM criterion (reduced food intake), respectively. It also includes CRP, which was used as a supporting proxy to determine the GLIM criterion of chronic disease/inflammation. In addition, anthropometric measures (body weight and waist circumference) are included in the model. Cut-off values were generated by the model for total weight loss, CRP, body weight, and waist circumference; with two different cut-off values for CRP depending on the decision path. These cut-offs are important because they help to refine the classification process.

The decision tree shown is an example of how ML data can be used to translate ML data into clinical applicability.

However, and again emphasizing the purely exemplary approach, some of the cut-off values generated by our decision tree are currently not suitable for practical use. A weight loss of less than 2.7% is barely detectable, but can be interpreted as weight loss in general being important, which also reflects the results of Yin et al. (40). Moreover, other parameters such as waist circumference or body weight are not yet related to the sex or height and may be strongly influenced by ascites, which was present in 21% of our patients (not shown). Although the overall aim was to identify common features of malnutrition in different chronic gastrointestinal diseases, we are aware that an individualized assessment is still necessary, and especially in decompensated LC patients with ascites, malnutrition assessment remains challenging.

## Limitations and outlook

The data in this study were collected from a smaller cohort of participants (*n* = 314), but include many features related to the nutritional status that were measured and recorded prospectively. The study was based on a cohort of controls and patients with different gastrointestinal diseases, including a high proportion of LC patients with ascites and edema. Therefore, our patient population is slightly biased toward liver disease. An imbalance in the proportion of attributes, e.g., male and female populations, could potentially lead to bias effects in the prognosis of the models. However, imbalanced attributes are common in clinical data and reflect real-world population characteristics. Our models were evaluated by cross-validation across multiple performance metrics, indicating that they capture meaningful relationships despite attribute imbalance. For the models used in this study, we found no sex bias in the prediction of malnutrition. In addition, the GLIM criteria were used to diagnose malnutrition, which served as the label for the classification task. This self-fulfilling design of the classification task may have biased the reported importance of the features. To overcome this, the GLIM criteria and associated features were excluded in a further approach with additional information gain.

Although the results of our models require further validation, our study provides a robust foundation for more in-depth research in this area by employing ML methods to classify malnutrition in chronic gastrointestinal diseases. Future research could address the pathomechanisms and possible different states of nutrition and malnutrition in chronic gastrointestinal diseases, as well as the impact of our models on the prognosis of patients belonging to different clusters.

## Conclusion

In this study, we employed ML approaches to identify the key features of malnutrition in patients with chronic gastrointestinal diseases. Among the extensive set of clinical features related to nutritional status, several GLIM criteria, as well as other features, were identified as important for the diagnosis of malnutrition. These include total weight loss, reduced food intake, inflammatory markers, hip and upper arm circumference, SMMI, phase angle, and clinical laboratory values of albumin, prealbumin and pseudocholinesterase. These results support the clinical applicability of the GLIM criteria in patients suffering from different gastrointestinal diseases and thus contribute to their validation. Furthermore, the diagnostic assessment of malnutrition may be improved by the implementation of additional criteria, such as anthropometric parameters, as well as classical laboratory values and possibly the use of decision trees. Prospective validation studies and future research are needed to gain further insight into the pathophysiology and prognosis of malnutrition in chronic gastrointestinal diseases.

## Data Availability

The original contributions presented in the study are included in the article/Supplementary material, further inquiries can be directed to the corresponding authors.
