# Supplementary material for: Identification of key factors for malnutrition diagnosis in chronic gastrointestinal diseases using machine learning underscores the importance of GLIM criteria as well as additional parameters
Source: Front Nutr. 2024 Dec 12;11:1479501. doi: 10.3389/fnut.2024.1479501 (PMC11670747; doi:10.3389/fnut.2024.1479501)
Supplement: Supplementary file 1 [file Table_1.DOCX]

# Supplementary material

**Table S1**: Features and encoding. From the originally curated features (n=230), n=135 features were selected for further ML analyses (marked in bold).

| Features (n=230) | Encoding |
| --- | --- |
| Recruitment Date |  |
| Examination Date |  |
| **Gastrointestinal Disease** | Healthy = 0, Controls = 1, SB/II= 2, CP = 3, LC = 4 |
| Child-Pugh-Turcotte Score (only for LC) | A = 1, B = 2, C = 3 |
| Numeric Rating Scale of Pain (only for CP) |  |
| COPPS (only for CP) | A = 1, B = 2, C = 3 |
| SB/II Type | I = 1, II = 2, III = 3 |
| Oral autonomy (only for SB/II) | primary = 1, secondary = 2 |
| Etiology of disease |  |
| Birth year |  |
| **Age** |  |
| **Sex** | male = 0, female = 1 |
| Study center |  |
| ESPEN Malnutrition diagnosis | malnourished = True, not malnourished = False |
| GLIM Malnutition diagnosis | malnourished = True, not malnourished = False |
| **GLIM_CRP (GLIM Malnutrition diagnosis implementing CRP)** | malnourished = True, not malnourished = False |
| Sarcopenia diagnosis (EWGSOP) | sarcopenic = True, not sarcopenic = False |
| **Sarcopenia (EWGSOP2, revised diagnosis)** | sarcopenic = True, not sarcopenic = False |
| Registered by |  |
| Treatment environment |  |
| Weight loss last 3 months | yes = True, no = False |
| Weight loss last 3 months (kg) |  |
| Weight loss > 5 %? | yes = True, no = False |
| Weight loss > 5 % (months) |  |
| Weight loss > 10 %? | yes = True, no = False |
| Weight loss > 10 % (months) |  |
| Weight loss since diagnosis | yes = True, no = False |
| Total weight loss (kg) |  |
| **Total weight loss (%)** |  |
| Food intake of the last week | < 25 % = 1, 25 - 50 % = 2, 50 - 75 % = 3, unchanged = 4 |
| Energy intake < 50 % > 1 week | yes = True, no = False |
| Reduced food intake > 2 weeks | yes = True, no = False |
| Malabsorptive disease | yes = True, no = False |
| **Reduced food intake (GLIM criterion)** | yes = True, no = False |
| Disease/inflammation | yes = True, no = False |
| **Disease/inflammation (based on CRP level)** | yes = True, no = False |
| Stage of disease | none = 0, mild = 1, moderate = 2 |
| Points NRS-2002 |  |
| **Malnutrition risk (NRS-2002 or RFH-NPT for LC)** | yes = True, no = False |
| **Edema** | yes = True, no = False |
| **Ascites** | yes = True, no = False |
| Points RFH-NPT (only for LC) |  |
| Malnutrition risk RFH-NPT (only for LC) | low = 1, moderate = 2, high = 3 |
| BMI < 18.5 kg/m^2^ | yes = True, no = False |
| Weight loss > 5 % in last 3 months or > 10 % indefinite of time | yes = True, no = False |
| Weight loss > 5 % in last 6 months or > 10 % indefinite of time | yes = True, no = False |
| BMI < 20 kg/m^2^ (< 70 y.) or < 22 kg/m^2^ (> 70 y.) | yes = True, no = False |
| FFMI < 15 kg/m^2^ (f) or < 17 kg/m^2^ (m) | yes = True, no = False |
| **Alcohol consumption** | never = 0, not anymore = 1, occasionally = 2, regularly = 3 |
| Not anymore since (alcohol) |  |
| Nicotine consumption | no = 0, Former smoker = 1, yes = 2 |
| Cigarettes/day |  |
| Since … years |  |
| **Pack Years (cigarettes)** |  |
| **Upper arm circumference** |  |
| Triceps skin fold 1 |  |
| Triceps skin fold 2 |  |
| Triceps skin fold 3 |  |
| **Triceps skin fold average** |  |
| **Waist circumference** |  |
| **Hip circumference** |  |
| Waist hip ratio |  |
| Device for BIA measurement |  |
| No food intake for > 4 h |  |
| Last alcohol consumption > 24 h ago |  |
| Physical exertion > 12 h ago |  |
| Empty bladder |  |
| **Body mass index (BMI)** |  |
| Relative fat mass |  |
| Absolute fat mass |  |
| Fat-free mass |  |
| Skeletal muscle mass |  |
| SMM (torso) |  |
| SMM (RL) |  |
| SMM (LL) |  |
| SMM (LA) |  |
| SMM (RA) |  |
| **Total Body Water** |  |
| **Extracellular water** |  |
| Waist circumference BIA |  |
| **Body weight** |  |
| **Body height** |  |
| **Resting energy expenditure (BIA)** |  |
| **Resistance (BIVA R)** |  |
| **Reactance (BIVA Xc)** |  |
| BIVA Z(R) |  |
| BIVA Z(Xc) |  |
| **Phase angle** |  |
| SDS PhaseAngle |  |
| Percentile PhaseAngle |  |
| ECW by TBW |  |
| **Fat mass index (FMI)** |  |
| Relative fat free mass (FFM) |  |
| **Fat free mass index (FFMI)** |  |
| Relative skeletal muscle mass (SMM) |  |
| **Skeletal muscle mass index (SSMI)** |  |
| Hand grip strength 1 |  |
| Hand grip strength 2 |  |
| Hand grip strength 3 |  |
| **Maximum handgrip strength** |  |
| Time for 4 m walking distance |  |
| **Gait speed** |  |
| **Marital status** | unmarried = 0, married, living together = 1, married, living seperately = 3, divorced = 3, widowed = 4 |
| **Living with partner** | yes = True, no = False |
| **No. of persons in household** |  |
| **No. of own children** |  |
| **Highest degree obtained** | without general school leaving certificate = 0, basic secondary school leaving certificate = 1, polytechnic secondary school leaving certificate = 2, intermediate secondary school leaving certificate = 3, university entrance qualification (Fachabitur, Abitur) = 4, Bachelor's degree = 5, Master's degree, diploma, engineering degree = 6, Completion of doctoral studies = 7 |
| **Employment** | not employed, unemployed = 0, not employed, early retirement, retirement = 1, Part-time or hourly employed (less than 15 hours/week) = 2, Part-time employed (more than 15 hours/week) = 3, Full-time employment = 4 |
| **Hemoglobin** |  |
| Hematocrit |  |
| Erythrocytes |  |
| **MCV** |  |
| MCH |  |
| **MCHC** |  |
| **Leukocytes** |  |
| **Thrombocytes** |  |
| Average thrombocyte volume |  |
| Thromboplastin time (Quick) |  |
| Thromboplastin time (s) |  |
| **International normalized ratio (INR)** |  |
| **Sodium blood** |  |
| **Potassium blood** |  |
| **Calcium blood** |  |
| **Magnesium blood** |  |
| **Phosphate blood** |  |
| **ASAT (GOT)** |  |
| **ALAT (GPT)** |  |
| **Gamma-GT** |  |
| **Alkaline Phosphatase** |  |
| **Pseudocholinesterase** |  |
| **Cholesterol blood** |  |
| **Triglycerides blood** |  |
| **Albumin** |  |
| **CRP** |  |
| **Prealbumin** |  |
| **Bilirubin total** |  |
| **Creatinine** |  |
| **Glucose blood** |  |
| **Uric acid** |  |
| **Urea** |  |
| **Zinc blood** |  |
| **Insulin** |  |
| **IGF 1** |  |
| **IL 1 beta** |  |
| **IL-6** |  |
| **TNF alpha** |  |
| Aldosterone |  |
| Copeptin |  |
| Renin |  |
| Bilirubin, direct |  |
| Bilirubin, indirect |  |
| Hemoglobin A1C (%) |  |
| Hemoglobin A1C (mmol/mol Hb) |  |
| Vigorous metabolic equivalent of task (METs)/week |  |
| Moderate METs/week |  |
| Walking METs/week |  |
| Total METs/week |  |
| **Physical activity level** | low = 1, moderate = 2, high = 3 |
| Category score |  |
| Basal metabolic rate (MJ)^[[1]](#footnote-1)^ |  |
| Basal metabolic rate (kcal)^1^ |  |
| **kcal**^1^ |  |
| **Protein**^1^ |  |
| **Fat total**^1^ |  |
| **Fat - saturated fatty acids (SFA)**^1^ |  |
| **Fat - monounsaturated fatty acids (MUFA)**^1^ |  |
| **Fat - polyunsaturated fatty acids** (**PUFA)**^1^ |  |
| **Fat - cholesterol**^1^ |  |
| **Carbohydrates**^1^ |  |
| **Alcohol**^1^ |  |
| **Fiber**^1^ |  |
| **Water**^1^ |  |
| **Sodium intake**^1^ |  |
| **Potassium intake**^1^ |  |
| **Calcium intake**^1^ |  |
| **Phosphate intake**^1^ |  |
| **Magnesium intake**^1^ |  |
| **Iron intake**^1^ |  |
| **Zinc intake**^1^ |  |
| **Vitamin A intake**^1^ |  |
| **Vitamin E intake**^1^ |  |
| **Vitamin B1 intake**^1^ |  |
| **Vitamin B2 intake**^1^ |  |
| **Vitamin B6 intake**^1^ |  |
| **Vitamin B12 intake**^1^ |  |
| **Vitamin C intake**^1^ |  |
| **Folic acid intake**^1^ |  |
| **Anxiety score^[[2]](#footnote-2)^** |  |
| **Depression score^2^** |  |
| Anxiety^2^ | normal = 0, borderline abnormal = 1, abnormal = 2 |
| Depression^2^ | normal = 0, borderline abnormal = 1, abnormal = 2 |
| Fatigue Symptome Score (FSS), average^[[3]](#footnote-3)^ |  |
| **Fatigue (FSS, dichotom)^3^** | none = False, fatigue = True |
| **Loneliness score^[[4]](#footnote-4)^** |  |
| Loneliness^4^ | least lonely = False, very lonely = True |
| **Food Frequency Score^5^** |  |
| **Food Frequency Score Pattern^5^** | unfavourable = 0, intermediary = 1, recommended = 2 |
| **Consumption of meat^[[5]](#footnote-5)^** | rare = 1, medium = 2, frequent = 3 |
| **Consumption of sausages and cold meat^5^** | rare = 1, medium = 2, frequent = 3 |
| **Consumption of fast food^5^** | rare = 1, medium = 2, frequent = 3 |
| **Consumption of poultry meat^5^** | rare = 1, medium = 2, frequent = 3 |
| **Consumption of fish^5^** | rare = 1, medium = 2, frequent = 3 |
| **Consumption of cooked potatoes^5^** | rare = 1, medium = 2, frequent = 3 |
| **Consumption of potato products^5^** | rare = 1, medium = 2, frequent = 3 |
| **Consumption of pasta^5^** | rare = 1, medium = 2, frequent = 3 |
| **Consumption of rice^5^** | rare = 1, medium = 2, frequent = 3 |
| **Consumption of fresh vegetables^5^** | rare = 1, medium = 2, frequent = 3 |
| **Consumption of cooked vegetables^5^** | rare = 1, medium = 2, frequent = 3 |
| **Consumption of fruit^5^** | rare = 1, medium = 2, frequent = 3 |
| **Consumption of white flour products^5^** | rare = 1, medium = 2, frequent = 3 |
| **Consumption of whole grain products^5^** | rare = 1, medium = 2, frequent = 3 |
| **Consumption of brown bread^5^** | rare = 1, medium = 2, frequent = 3 |
| **Consumption of muesli^5^** | rare = 1, medium = 2, frequent = 3 |
| **Consumption of butter^5^** | rare = 1, medium = 2, frequent = 3 |
| **Consumption of margarine^5^** | rare = 1, medium = 2, frequent = 3 |
| **Consumption of olive oil^5^** | rare = 1, medium = 2, frequent = 3 |
| **Consumption of vegetable oils^5^** | rare = 1, medium = 2, frequent = 3 |
| **Consumption of cream^5^** | rare = 1, medium = 2, frequent = 3 |
| **Consumption of curd/yogurt^5^** | rare = 1, medium = 2, frequent = 3 |
| **Consumption of low fat dairy products^5^** | rare = 1, medium = 2, frequent = 3 |
| **Consumption of dairy products^5^** | rare = 1, medium = 2, frequent = 3 |
| **Consumption of cheese^5^** | rare = 1, medium = 2, frequent = 3 |
| **Consumption of eggs^5^** | rare = 1, medium = 2, frequent = 3 |
| **Consumption of pastries^5^** | rare = 1, medium = 2, frequent = 3 |
| **Consumption of confectionary^5^** | rare = 1, medium = 2, frequent = 3 |
| **Consumption of salty snacks^5^** | rare = 1, medium = 2, frequent = 3 |
| **Consumption of fruit/vegetable juice^5^** | rare = 1, medium = 2, frequent = 3 |
| **Consumption of soft drinks^5^** | rare = 1, medium = 2, frequent = 3 |
| **Consumption of water^5^** | rare = 1, medium = 2, frequent = 3 |
| **Consumption of low alcohol beer^5^** | rare = 1, medium = 2, frequent = 3 |

**Table S2**: Average performance of the trained models classifying malnutrition using all (n=134) features. Performance was evaluated for 100 x 10-fold cross-validation iterations (n=1000). The highest value for each performance metric across all algorithms is highlighted. Data are shown as mean ± standard deviation. KNN = K-Nearest Neighbors, SVM = Support Vector Machine, AdaBoost = Adaptive Boosting, LGBM = Light Gradient Boosting Machine, XGBoost = eXtreme Gradient Boosting, ROC AUC = area under the receiver operating characteristic.

|  | **Decision Tree** | **KNN** | **SVM** | **Logistic Regression** | **Naive Bayes** | **Random Forest** | **AdaBoost** | **LGBM** | **XGBoost** |
| --- | --- | --- | --- | --- | --- | --- | --- | --- | --- |
| Accuracy | 0.898 ± 0.059 | 0.801 ± 0.058 | 0.837 ± 0.063 | 0.863 ± 0.059 | 0.793 ± 0.073 | 0.888 ± 0.055 | 0.915 ± 0.050 | **0.920 ± 0.049** | 0.907 ± 0.048 |
| Precision | 0.838 ± 0.108 | 0.854 ± 0.141 | 0.741 ± 0.109 | 0.751 ± 0.099 | 0.696 ± 0.123 | 0.830 ± 0.104 | **0.888 ± 0.094** | 0.880 ± 0.092 | 0.876 ± 0.093 |
| Sensitivity | 0.875 ± 0.107 | 0.491 ± 0.152 | 0.807 ± 0.123 | **0.903 ± 0.096** | 0.696 ± 0.140 | 0.849 ± 0.107 | 0.863 ± 0.109 | 0.888 ± 0.101 | 0.849 ± 0.112 |
| Specificity | 0.909 ± 0.070 | **0.954 ± 0.046** | 0.852 ± 0.077 | 0.843 ± 0.078 | 0.841 ± 0.083 | 0.907 ± 0.065 | 0.941 ± 0.055 | 0.935 ± 0.054 | 0.935 ± 0.053 |
| ROC AUC | 0.932 ± 0.056 | 0.867 ± 0.061 | 0.915 ± 0.049 | 0.953 ± 0.032 | 0.851 ± 0.072 | 0.955 ± 0.033 | 0.970 ± 0.030 | **0.971 ± 0.027** | 0.968 ± 0.029 |
| Average precision score | 0.859 ± 0.103 | 0.761 ± 0.100 | 0.844 ± 0.095 | 0.914 ± 0.059 | 0.717 ± 0.121 | 0.923 ± 0.058 | **0.953 ± 0.043** | 0.951 ± 0.048 | 0.949 ± 0.043 |
| F1 score | 0.850 ± 0.085 | 0.608 ± 0.139 | 0.766 ± 0.090 | 0.815 ± 0.076 | 0.688 ± 0.110 | 0.833 ± 0.082 | 0.870 ± 0.079 | **0.879 ± 0.075** | 0.856 ± 0.078 |
| Balanced accuracy | 0.892 ± 0.064 | 0.722 ± 0.078 | 0.830 ± 0.070 | 0.873 ± 0.059 | 0.768 ± 0.082 | 0.878 ± 0.062 | 0.902 ± 0.060 | **0.912 ± 0.057** | 0.892 ± 0.060 |
| Cohen’s Kappa score | 0.773 ± 0.128 | 0.492 ± 0.159 | 0.642 ± 0.137 | 0.709 ± 0.121 | 0.535 ± 0.160 | 0.749 ± 0.122 | 0.807 ± 0.115 | **0.820 ± 0.110** | 0.787 ± 0.112 |

**Table S3**: Average performance of the trained models classifying malnutrition leaving out GLIM diagnosis criteria (total weight loss, BMI, FFMI, C-reactive protein, reduced food intake, chronic disease/inflammation, malnutrition risk; resulting in n=127 features). Performance was evaluated for 100 x 10-fold cross-validation iterations (n=1000). The highest value for each performance metric across all algorithms is highlighted. Data are shown as mean ± standard deviation. KNN = K-Nearest Neighbors, SVM = Support Vector Machine, AdaBoost = Adaptive Boosting, LGBM = Light Gradient Boosting Machine, XGBoost = eXtreme Gradient Boosting, ROC AUC = area under the receiver operating characteristic.

|  | **Decision Tree** | **KNN** | **SVM** | **Logistic Regression** | **Naive Bayes** | **Random Forest** | **AdaBoost** | **LGBM** | **XGBoost** |
| --- | --- | --- | --- | --- | --- | --- | --- | --- | --- |
| Accuracy | 0.741 ± 0.075 | 0.748 ± 0.058 | 0.782 ± 0.070 | 0.745 ± 0.075 | 0.758 ± 0.074 | **0.816 ± 0.066** | 0.795 ± 0.067 | 0.803 ± 0.067 | 0.796 ± 0.066 |
| Precision | 0.581 ± 0.089 | **0.737 ± 0.179** | 0.669 ± 0.115 | 0.596 ± 0.099 | 0.656 ± 0.136 | 0.733 ± 0.117 | 0.708 ± 0.124 | 0.704 ± 0.117 | 0.715 ± 0.126 |
| Sensitivity | **0.869 ± 0.119** | 0.390 ± 0.144 | 0.709 ± 0.135 | 0.770 ± 0.124 | 0.603 ± 0.147 | 0.721 ± 0.132 | 0.679 ± 0.144 | 0.731 ± 0.136 | 0.667 ± 0.146 |
| Specificity | 0.677 ± 0.111 | **0.926 ± 0.056** | 0.818 ± 0.082 | 0.732 ± 0.095 | 0.834 ± 0.084 | 0.862 ± 0.074 | 0.853 ± 0.078 | 0.839 ± 0.080 | 0.860 ± 0.076 |
| ROC AUC | 0.820 ± 0.065 | 0.801 ± 0.074 | 0.852 ± 0.065 | 0.838 ± 0.066 | 0.811 ± 0.081 | **0.884 ± 0.057** | 0.861 ± 0.063 | 0.871 ± 0.062 | 0.865 ± 0.062 |
| Average precision score | 0.621 ± 0.099 | 0.654 ± 0.108 | 0.737 ± 0.114 | 0.736 ± 0.107 | 0.669 ± 0.126 | **0.791 ± 0.105** | 0.758 ± 0.109 | 0.773 ± 0.108 | 0.769 ± 0.107 |
| F1 score | 0.690 ± 0.082 | 0.496 ± 0.143 | 0.681 ± 0.103 | 0.667 ± 0.092 | 0.619 ± 0.120 | **0.720 ± 0.102** | 0.684 ± 0.109 | 0.709 ± 0.102 | 0.680 ± 0.111 |
| Balanced accuracy | 0.773 ± 0.071 | 0.658 ± 0.075 | 0.764 ± 0.078 | 0.751 ± 0.077 | 0.719 ± 0.084 | **0.792 ± 0.075** | 0.766 ± 0.078 | 0.785 ± 0.076 | 0.764 ± 0.078 |
| Cohen’s Kappa score | 0.485 ± 0.136 | 0.354 ± 0.159 | 0.517 ± 0.152 | 0.467 ± 0.148 | 0.444 ± 0.168 | **0.583 ± 0.147** | 0.534 ± 0.153 | 0.562 ± 0.148 | 0.532 ± 0.153 |

**Table S4**: Ranking of SHAP feature importance values from the LGBM including all features.

| **Ranking** | **Feature** | **mean ± SD** |
| --- | --- | --- |
| 1 | Total weight loss | 2.016 ± 2.168 |
| 2 | Reduced food intake | 1.702 ± 1.793 |
| 3 | C-reactive protein | 0.558 ± 0.616 |
| 4 | Body weight | 0.522 ± 0.552 |
| 5 | Waist circumference | 0.370 ± 0.529 |
| 6 | Albumin | 0.368 ± 0.404 |
| 7 | Disease/inflammation | 0.365 ± 0.435 |
| 8 | Resistance | 0.335 ± 0.364 |
| 9 | TNF alpha | 0.221 ± 0.248 |
| 10 | Physical activity level | 0.221 ± 0.274 |
| 11 | Iron intake | 0.191 ± 0.205 |
| 12 | Hip circumference | 0.177 ± 0.188 |
| 13 | Fatigue (FSS, dichotom) | 0.167 ± 0.201 |
| 14 | Malnutrition risk | 0.155 ± 0.202 |
| 15 | MCV | 0.148 ± 0.157 |
| 16 | IL-6 | 0.147 ± 0.183 |
| 17 | Fat free mass index | 0.144 ± 0.200 |
| 18 | Creatinine | 0.137 ± 0.152 |
| 19 | Urea | 0.119 ± 0.131 |
| 20 | International normalized ratio (INR) | 0.111 ± 0.149 |
| 21 | Calcium blood | 0.108 ± 0.119 |
| 22 | Zinc blood | 0.104 ± 0.141 |
| 23 | Thrombocytes | 0.088 ± 0.097 |
| 24 | Skeletal muscle mass index | 0.082 ± 0.096 |
| 25 | Gamma-GT | 0.078 ± 0.096 |
| 26 | ASAT (GOT) | 0.067 ± 0.077 |
| 27 | Alcohol | 0.065 ± 0.079 |
| 28 | Potassium blood | 0.053 ± 0.059 |
| 29 | Pack years (cigarettes) | 0.052 ± 0.094 |
| 30 | Fat - cholesterol | 0.049 ± 0.054 |
| 31 | Pseudocholinesterase | 0.048 ± 0.063 |
| 32 | Fat total | 0.047 ± 0.066 |
| 33 | Prealbumin | 0.045 ± 0.051 |
| 34 | Depression score | 0.044 ± 0.056 |
| 35 | Body mass index | 0.042 ± 0.048 |
| 36 | Gastrointestinal disease | 0.042 ± 0.048 |
| 37 | Consumption of cooked potatoes | 0.042 ± 0.048 |
| 38 | Uric acid | 0.041 ± 0.051 |
| 39 | Anxiety score | 0.038 ± 0.044 |
| 40 | IGF 1 | 0.033 ± 0.038 |
| 41 | Leukocytes | 0.033 ± 0.036 |
| 42 | Fat mass index | 0.032 ± 0.037 |
| 43 | Water | 0.030 ± 0.036 |
| 44 | Folic acid | 0.029 ± 0.032 |
| 45 | Carbohydrates | 0.027 ± 0.032 |
| 46 | Consumption of cooked vegetables | 0.027 ± 0.031 |
| 47 | Extracellular water | 0.026 ± 0.029 |
| 48 | Gait speed | 0.026 ± 0.032 |
| 49 | Calcium intake | 0.025 ± 0.030 |
| 50 | IL 1 beta | 0.025 ± 0.034 |
| 51 | FFS pattern | 0.025 ± 0.030 |
| 52 | Magnesium blood | 0.024 ± 0.031 |
| 53 | Fat - monounsaturated fatty acids (MUFA) | 0.024 ± 0.035 |
| 54 | Phase angle | 0.023 ± 0.025 |
| 55 | Maximum handgrip strength | 0.023 ± 0.029 |
| 56 | Glucose blood | 0.023 ± 0.025 |
| 57 | Bilirubin total | 0.022 ± 0.027 |
| 58 | Zinc intake | 0.022 ± 0.027 |
| 59 | Triglycerides blood | 0.021 ± 0.031 |
| 60 | Triceps skin fold average | 0.021 ± 0.027 |
| 61 | Phosphate blood | 0.019 ± 0.024 |
| 62 | ALAT (GPT) | 0.019 ± 0.023 |
| 63 | Vitamin A | 0.018 ± 0.024 |
| 64 | Living with partner | 0.018 ± 0.021 |
| 65 | Consumption of fruit/vegetable juice | 0.018 ± 0.020 |
| 66 | Vitamin B1 | 0.017 ± 0.026 |
| 67 | Consumption of low fat dairy products | 0.016 ± 0.018 |
| 68 | Alkaline Phosphatase | 0.016 ± 0.019 |
| 69 | Consumption of cream | 0.014 ± 0.016 |
| 70 | Sodium intake | 0.013 ± 0.015 |
| 71 | Reactance | 0.013 ± 0.015 |
| 72 | Phosphate intake | 0.012 ± 0.014 |
| 73 | Vitamin B2 | 0.012 ± 0.014 |
| 74 | Fiber | 0.012 ± 0.015 |
| 75 | MCHC | 0.012 ± 0.013 |
| 76 | Magnesium intake | 0.012 ± 0.013 |
| 77 | Consumption of olive oil | 0.011 ± 0.013 |
| 78 | Fat - saturated fatty acids (SFA) | 0.011 ± 0.014 |
| 79 | Age | 0.010 ± 0.012 |
| 80 | Resting energy expenditure (BIA) | 0.010 ± 0.012 |
| 81 | Hemoglobin | 0.009 ± 0.013 |
| 82 | Vitamin E | 0.009 ± 0.011 |
| 83 | Protein | 0.009 ± 0.012 |
| 84 | Consumption of whole grain products | 0.009 ± 0.011 |
| 85 | kcal | 0.009 ± 0.011 |
| 86 | Insulin | 0.008 ± 0.010 |
| 87 | Highest degree obtained | 0.007 ± 0.009 |
| 88 | No. of own children | 0.007 ± 0.007 |
| 89 | Consumption of brown bread | 0.006 ± 0.007 |
| 90 | Total body water | 0.006 ± 0.008 |
| 91 | Cholesterol blood | 0.005 ± 0.007 |
| 92 | Body height | 0.005 ± 0.007 |
| 93 | Marital status | 0.005 ± 0.007 |
| 94 | Vitamin C | 0.005 ± 0.005 |
| 95 | Upper arm circumference | 0.004 ± 0.006 |
| 96 | Consumption of curd/yogurt | 0.004 ± 0.005 |
| 97 | Fat - polyunsaturated fatty acids (PUFA) | 0.004 ± 0.005 |
| 98 | Sodium blood | 0.004 ± 0.005 |
| 99 | Alcohol consumption | 0.004 ± 0.005 |
| 100 | Consumption of margarine | 0.004 ± 0.004 |
| 101 | Potassium intake | 0.003 ± 0.004 |
| 102 | Vitamin B6 | 0.003 ± 0.004 |
| 103 | Consumption of potato products | 0.003 ± 0.004 |
| 104 | Consumption of eggs | 0.003 ± 0.003 |
| 105 | Consumption of vegetable oils | 0.003 ± 0.003 |
| 106 | Vitamin B12 | 0.002 ± 0.003 |
| 107 | Sex | 0.002 ± 0.003 |
| 108 | Food Frequency Score | 0.002 ± 0.003 |
| 109 | Consumption of soft drinks | 0.002 ± 0.002 |
| 110 | Loneliness score | 0.002 ± 0.002 |
| 111 | Consumption of muesli | 0.002 ± 0.002 |
| 112 | Consumption of fresh vegetables | 0.002 ± 0.003 |
| 113 | Consumption of white flour products | 0.002 ± 0.002 |
| 114 | Consumption of fish | 0.002 ± 0.002 |
| 115 | Employment | 0.001 ± 0.002 |
| 116 | Consumption of dairy products | 0.001 ± 0.002 |
| 117 | Consumption of pastries | 0.001 ± 0.002 |
| 118 | Consumption of rice | 0.001 ± 0.001 |
| 119 | Consumption of pasta | 0.001 ± 0.001 |
| 120 | Edema | 0.001 ± 0.002 |
| 121 | Consumption of poultry meat | 0.001 ± 0.001 |
| 122 | Consumption of meat | 0.000 ± 0.001 |
| 123 | Consumption of confectionary | 0.000 ± 0.001 |
| 124 | Consumption of butter | 0.000 ± 0.001 |
| 125 | Consumption of salty snacks | 0.000 ± 0.001 |
| 126 | No. of persons in household | 0.000 ± 0.001 |
| 127 | Ascites | 0.000 ± 0.000 |
| 128 | Consumption of cheese | 0.000 ± 0.001 |
| 129 | Consumption of low alcohol beer | 0.000 ± 0.000 |
| 130 | Consumption of fast food | 0.000 ± 0.000 |
| 131 | Consumption of sausages and cold meat | 0.000 ± 0.000 |
| 132 | Consumption of fruit | 0.000 ± 0.000 |
| 133 | Sarcopenia | 0.000 ± 0.000 |
| 134 | Consumption of water | 0.000 ± 0.000 |

**Table S5**: Ranking of SHAP feature importance values from the Random Forest model leaving out the GLIM malnutrition diagnosis criteria and associated features malnutrition risk and C-reactive protein.

| **Ranking** | **Feature** | **mean ± SD** |
| --- | --- | --- |
| 1 | Gastrointestinal disease | 0.045 ± 0.053 |
| 2 | Albumin | 0.031 ± 0.033 |
| 3 | Hip circumference | 0.021 ± 0.021 |
| 4 | Body weight | 0.019 ± 0.020 |
| 5 | Pseudocholinesterase | 0.018 ± 0.019 |
| 6 | Prealbumin | 0.018 ± 0.018 |
| 7 | Phase angle | 0.017 ± 0.018 |
| 8 | Upper arm circumference | 0.017 ± 0.020 |
| 9 | Skeletal muscle mass index | 0.017 ± 0.020 |
| 10 | IL-6 | 0.015 ± 0.015 |
| 11 | TNF alpha | 0.015 ± 0.015 |
| 12 | Plasma sodium | 0.014 ± 0.015 |
| 13 | Resistance | 0.011 ± 0.012 |
| 14 | Triceps skin fold | 0.011 ± 0.014 |
| 15 | Alkaline Phosphatase | 0.011 ± 0.011 |
| 16 | Zinc blood | 0.010 ± 0.010 |
| 17 | Bilirubin | 0.008 ± 0.008 |
| 18 | Fat mass index | 0.008 ± 0.010 |
| 19 | International normalized ratio (INR) | 0.007 ± 0.009 |
| 20 | Uric acid | 0.007 ± 0.008 |
| 21 | Potassium blood | 0.007 ± 0.007 |
| 22 | Waist circumference | 0.007 ± 0.008 |
| 23 | Creatinine | 0.006 ± 0.007 |
| 24 | Hemoglobin | 0.006 ± 0.006 |
| 25 | Alcohol | 0.006 ± 0.006 |
| 26 | Resting energy expenditure (BIA) | 0.006 ± 0.007 |
| 27 | Depression score | 0.005 ± 0.006 |
| 28 | Gait speed | 0.004 ± 0.005 |
| 29 | Total body water | 0.004 ± 0.005 |
| 30 | Leukocytes | 0.004 ± 0.004 |
| 31 | Maximum handgrip strength | 0.004 ± 0.005 |
| 32 | Iron intake | 0.004 ± 0.004 |
| 33 | Gamma-GT | 0.004 ± 0.004 |
| 34 | Fatigue (FSS, dichotom) | 0.003 ± 0.004 |
| 35 | ASAT (GOT) | 0.003 ± 0.003 |
| 36 | IGF 1 | 0.003 ± 0.003 |
| 37 | Ascites | 0.003 ± 0.003 |
| 38 | Urea | 0.003 ± 0.004 |
| 39 | Extracellular water | 0.003 ± 0.003 |
| 40 | Physical activity level | 0.003 ± 0.003 |
| 41 | Water | 0.003 ± 0.004 |
| 42 | Magnesium intake | 0.003 ± 0.003 |
| 43 | Highest degree obtained | 0.002 ± 0.002 |
| 44 | Fat - saturated fatty acids (SFA) | 0.002 ± 0.002 |
| 45 | Insulin | 0.002 ± 0.003 |
| 46 | Fat total | 0.002 ± 0.002 |
| 47 | Consumption of white flour products | 0.002 ± 0.003 |
| 48 | Cholesterol blood | 0.002 ± 0.002 |
| 49 | Glucose blood | 0.002 ± 0.002 |
| 50 | Thrombocytes | 0.002 ± 0.002 |
| 51 | MCV | 0.002 ± 0.002 |
| 52 | Carbohydrates | 0.002 ± 0.002 |
| 53 | Triglycerides blood | 0.002 ± 0.002 |
| 54 | Fat - monounsaturated fatty acids (MUFA) | 0.002 ± 0.002 |
| 55 | Magnesium blood | 0.001 ± 0.002 |
| 56 | kcal | 0.001 ± 0.002 |
| 57 | Age | 0.001 ± 0.002 |
| 58 | Consumption of olive oil | 0.001 ± 0.001 |
| 59 | Sarcopenia | 0.001 ± 0.002 |
| 60 | Folic acid | 0.001 ± 0.002 |
| 61 | Food Frequency Score | 0.001 ± 0.002 |
| 62 | Sodium intake | 0.001 ± 0.001 |
| 63 | ALAT (GPT) | 0.001 ± 0.002 |
| 64 | Alcohol consumption | 0.001 ± 0.001 |
| 65 | Employment | 0.001 ± 0.001 |
| 66 | Consumption of curd/yogurt | 0.001 ± 0.001 |
| 67 | Anxiety score | 0.001 ± 0.001 |
| 68 | Vitamin A | 0.001 ± 0.001 |
| 69 | Edema | 0.001 ± 0.001 |
| 70 | Calcium blood | 0.001 ± 0.001 |
| 71 | Pack years (cigarettes) | 0.001 ± 0.001 |
| 72 | Vitamin C | 0.001 ± 0.001 |
| 73 | Calcium intake | 0.001 ± 0.001 |
| 74 | Phosphate intake | 0.001 ± 0.001 |
| 75 | Vitamin B12 | 0.001 ± 0.001 |
| 76 | Phosphate blood | 0.001 ± 0.001 |
| 77 | Vitamin B2 | 0.001 ± 0.001 |
| 78 | IL 1 beta | 0.001 ± 0.001 |
| 79 | Fat - cholesterol | 0.001 ± 0.001 |
| 80 | Consumption of margarine | 0.001 ± 0.001 |
| 81 | Vitamin B1 | 0.001 ± 0.001 |
| 82 | Consumption of fruit/vegetable juice | 0.001 ± 0.001 |
| 83 | Consumption of fish | 0.001 ± 0.001 |
| 84 | Fiber | 0.001 ± 0.001 |
| 85 | Consumption of whole grain products | 0.001 ± 0.001 |
| 86 | Consumption of sausages and cold meat | 0.001 ± 0.001 |
| 87 | Consumption of brown bread | 0.001 ± 0.001 |
| 88 | Reactance | 0.001 ± 0.001 |
| 89 | Zinc intake | 0.001 ± 0.001 |
| 90 | Protein | 0.001 ± 0.001 |
| 91 | Loneliness score | 0.001 ± 0.002 |
| 92 | Consumption of pastries | 0.001 ± 0.001 |
| 93 | Potassium intake | 0.001 ± 0.001 |
| 94 | Vitamin E | 0.001 ± 0.001 |
| 95 | Body height | 0.001 ± 0.001 |
| 96 | Marital status | 0.001 ± 0.001 |
| 97 | Vitamin B6 | 0.001 ± 0.001 |
| 98 | Fat - polyunsaturated fatty acids (PUFA) | 0.000 ± 0.001 |
| 99 | MCHC | 0.000 ± 0.000 |
| 100 | Consumption of confectionary | 0.000 ± 0.000 |
| 101 | Consumption of muesli | 0.000 ± 0.001 |
| 102 | Consumption of cooked potatoes | 0.000 ± 0.000 |
| 103 | Consumption of vegetable oils | 0.000 ± 0.000 |
| 104 | Consumption of salty snacks | 0.000 ± 0.000 |
| 105 | Consumption of soft drinks | 0.000 ± 0.000 |
| 106 | Consumption of meat | 0.000 ± 0.000 |
| 107 | Consumption of potato products | 0.000 ± 0.000 |
| 108 | Consumption of fresh vegetables | 0.000 ± 0.000 |
| 109 | Consumption of cooked vegetables | 0.000 ± 0.000 |
| 110 | Consumption of cheese | 0.000 ± 0.001 |
| 111 | Consumption of pasta | 0.000 ± 0.000 |
| 112 | Living with partner | 0.000 ± 0.000 |
| 113 | Consumption of fast food | 0.000 ± 0.000 |
| 114 | FFS pattern | 0.000 ± 0.001 |
| 115 | Consumption of fruit | 0.000 ± 0.000 |
| 116 | Consumption of dairy products | 0.000 ± 0.000 |
| 117 | No. of own children | 0.000 ± 0.000 |
| 118 | Sex | 0.000 ± 0.000 |
| 119 | Consumption of low alcohol beer | 0.000 ± 0.000 |
| 120 | Consumption of cream | 0.000 ± 0.000 |
| 121 | No. of persons in household | 0.000 ± 0.000 |
| 122 | Consumption of rice | 0.000 ± 0.000 |
| 123 | Consumption of eggs | 0.000 ± 0.000 |
| 124 | Consumption of low fat dairy products | 0.000 ± 0.000 |
| 125 | Consumption of butter | 0.000 ± 0.000 |
| 126 | Consumption of poultry meat | 0.000 ± 0.000 |
| 127 | Consumption of water | 0.000 ± 0.000 |

1. Intake per day, calculated from DEGS food frequency questionnaire [↑](#footnote-ref-1)
2. from HADS-D questionnaire [↑](#footnote-ref-2)
3. from Fatigue Severity Scale [↑](#footnote-ref-3)
4. from De Jong Gierveld Scale [↑](#footnote-ref-4)
5. from SHIP food frequency questionnaire [↑](#footnote-ref-5)
